# Continuous-flow retro-Diels–Alder reaction: an efficient method for the preparation of pyrimidinone derivatives

**DOI:** 10.3762/bjoc.14.20

**Published:** 2018-02-01

**Authors:** Imane Nekkaa, Márta Palkó, István M Mándity, Ferenc Fülöp

**Affiliations:** 1 Institute of Pharmaceutical Chemistry, University of Szeged, Eötvös u. 6, H-6720 Szeged, Hungary,; 2 MTA-SZTE Stereochemistry Research Group, Hungarian Academy of Sciences, Eötvös u. 6, H-6720 Szeged, Hungary

**Keywords:** continuous-flow, desulfurisation, norbornene-fused heterocycles, pyrimidinones, retro-Diels–Alder reaction

## Abstract

The syntheses of various pyrimidinones as potentially bioactive products by means of the highly controlled continuous-flow retro-Diels–Alder reaction of condensed pyrimidinone derivatives are presented. Noteworthy, the use of this approach allowed us to rapidly screen a selection of conditions and quickly confirm the viability of preparing the desired pyrimidinones in short reaction times. Yields typically higher than those published earlier using conventional batch or microwave processes were achieved.

## Introduction

The continuous-flow (CF) technology has gained significant importance in modern synthetic chemistry [1–3] and becomes a core technology in the pharmaceutical, agrochemical, and fine chemical industries [4–5]. The use of this technology opens a new door to a quick optimization, acceleration [6], and easy scale-up with a wide and growing range of chemical transformations in combination with an inherently safe and green nature [7–12]. Advantageously, safety issues are complied with excellent mixing and heat transfer [7–14]. These allow the access to elevated temperatures and pressures accredited to superheating of organic solvents in a controlled and safe fashion [6,14–17]. The accurate tuning of residence time can further broaden the versatility of CF processes by governing the outcome of chemical reactions, determining the reaction rate and the conversion and by influencing product selectivities [17–19]. Thus, flow chemistry has long been selected to provide a simple means to use more rigorous reaction conditions and revisit difficult reactions that have been neglected in the past [21].

The retro-Diels–Alder (rDA) reaction has become an important tool for synthetic chemists in their search towards the synthesis and design of novel heterocyclic scaffolds. This pyrolytic dissociation arises when one or both fragments are notably stable [22]. The unsaturation present in the original starting material is produced in the DA addition, and the same atoms are involved in both the bond formation and cleavage steps [23–25]. The rDA process is an efficient technique for the introduction of a double bond into a heterocyclic skeleton [26] as well as for the enantiodivergent [27] and the enantiocontrolled [28] syntheses of heterocyclic compounds. The rDA products can be gained, due to a thermal [4 + 2]-cycloreversion, by distillation under reduced pressure [29], boiling in solvent [30–31], and applying microwave irradiation [32–35] or flash vacuum pyrolysis [35–36]. rDA reactions under mild conditions have been widely examined and discussed for the laboratory preparation of heteromonocycles or condensed-ring heterocycles [37–40]. However, the CF rDA method was introduced when Meyers’ group performed the preparation of a precursor intermediate for the construction of diverse tetracycline antibiotics [41]. Our aim in the present study was to synthesize functionalized pyrimidinone systems through rDA reactions. Many of these products are of high importance in drug design due to their diverse biological properties including antimicrobial, antiviral, antioxidant and antitumor activities. In addition, they are present in several natural frameworks [42–44].

We wanted to exploit the benefits of flow processing for reaction optimization and synthesis and develop novel sustainable synthetic methodologies with possible useful applications for the pharmaceutical industry. Our results show that the developed CF technology is superior to existing conventional batch technologies.

## Results and Discussion

The starting materials, i.e., fused tricyclic or tetracyclic pyrimidinones **1**–**8** have been previously prepared by literature methods [26,45–50]. Cyclization of the corresponding di-*exo*- or di*-endo*-amino acids or esters with ethyl *p*-chlorobenzimidate resulted in tricyclic pyrimidinones **1**, **2a** and **2b** [26,45–49]. Methanopyrrolo-, methanopyrido- and methanoazepino[2,1-*b*]quinazolinones **3**–**6** were prepared by ring enlargement of di*-exo*-norbornene-fused azetidinones with lactim ethers [50]. For the preparation of 2-thioxopyrimidinones **7**, **8a** and **8b**, the most common method is the reaction of the appropriate amino esters with phenyl isothiocyanate, followed by cyclization of the resulting thiourea with hydrogen chloride under reflux [45,49]. The starting materials were selected to comprise molecules where good (>80%), medium (70–80%) and no conversion was observed under batch rDA conditions. Batch reactions were carried out by the following ways: heating under neat conditions, refluxing in solvents having a high boiling point [chlorobenzene (CB) or 1,2-dichlorobenzene (DCB)], and under microwave (MW) conditions in DCB.

**Scheme 1:**
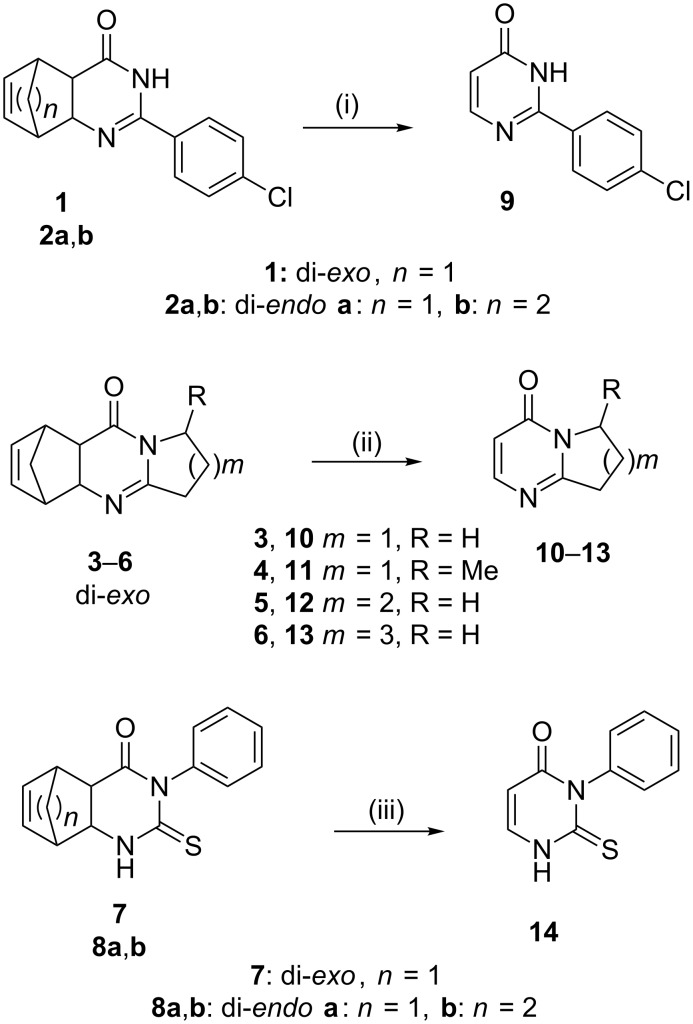
Flow synthesis for the preparation of fused pyrimidinones **9**–**14** by rDA reaction. Solvent and conditions (*F*_R_ is the flow rate): (i) MeCN, toluene, *F*_R_ = 0.5 mL min^−1^, 230–250 °C; (ii) MeOH, *F*_R_ = 0.5 mL min^−1^, 120–150 °C; (iii) MeCN, *F*_R_ = 0.5 mL min^−1^, 220–250 °C.

**Figure 1:**
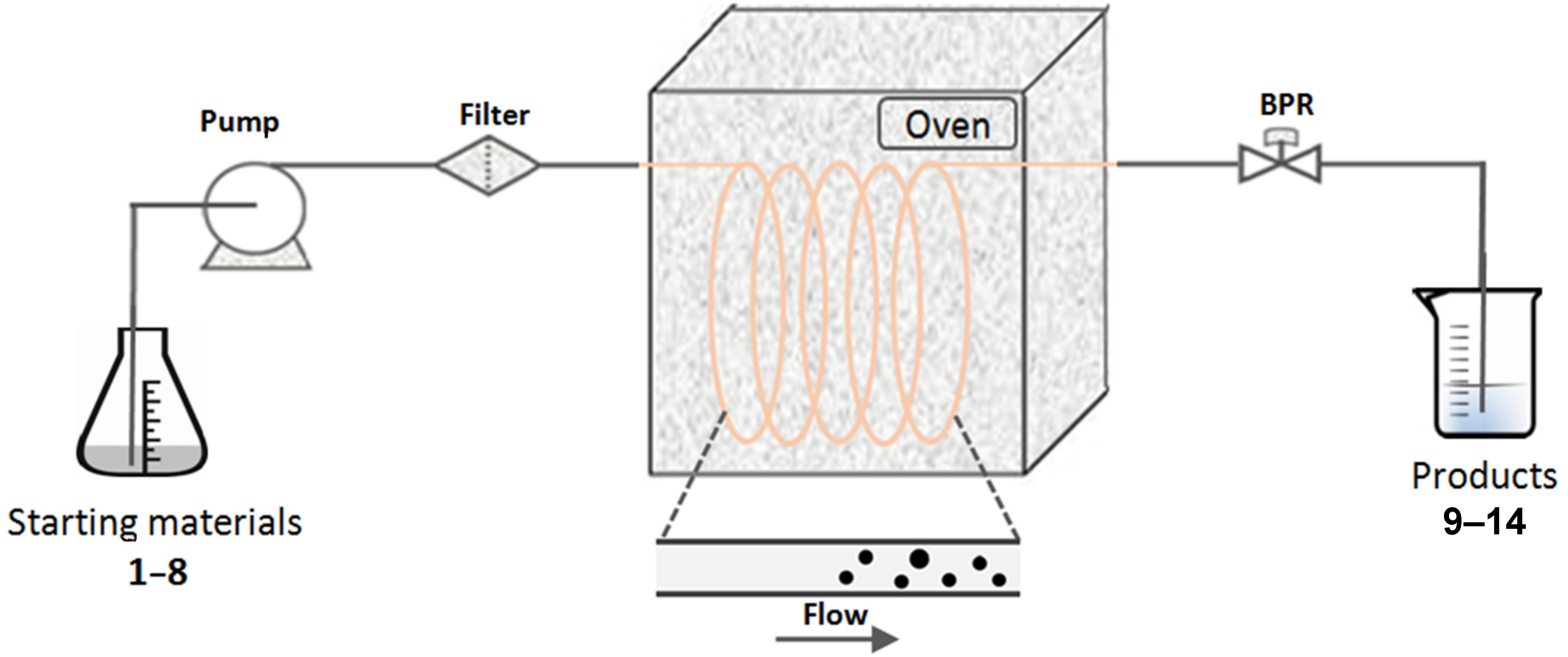
Schematic outline of the continuous-flow reactor.

In order to provide a rapid and efficient access to the desired pyrimidinones **9**–**14** (Scheme 1), we reinvestigated these rDA reactions by using another method involving flow chemistry. Therefore, a modular flow system was designed, equipped with a heated 304 stainless steel coil and an adjustable back-pressure regulator (0–300 bar) controlling the use of solvents under superheated conditions. The coil was heated in an oven to the desired temperature and solutions of the starting materials **1**–**8** were loaded into the reactor via a HPLC pump. Solvents were selected on the basis of the solubility of the starting materials. A schematic representation of the flow reactor setup is illustrated in Figure 1. Products **9**–**14** thus prepared were identified by means of HPLC–MS and NMR spectroscopic analysis. All physical and spectroscopic data of pyrimidinones **9**–**14** were identical with their literature data (Supporting Information File 1).

The rDA reaction is basically a thermally-driven process. Consequently, by careful reaction parameter optimization, a balance should be found between the desired rDA cycloreversion reaction and the unwanted thermal degradation of the rDA product. The conversion and yield of a reaction under CF conditions is influenced directly by the residence time and reaction temperature, which are crucial determining factors in flow chemistry [18–20]. Thus, these two parameters were fine-tuned for all of the starting materials. The residence time was set by the use of coils with different lengths. The pressure and flow rate of the reactions were kept at constant values of 10 bars and 0.5 mL min^−1^, respectively. The full reaction parameter optimization is shown only for compound **1** in Table 1.

**Table 1:** Reaction parameter optimization for the CF rDA reaction of **1**.

entry	temperature [°C]	residence time [min]	conversion [%]	degradation product [%]

1	200	10	64	–
2	210	10	82	–
3	220	10	83	–
4	230	10	86	0
5	240	10	100	7
6	250	10	100	18
7	230	15	100	0
8	230	30	100	13

The tricyclic di*-exo*-2-(4-chlorophenyl)tetrahydro-5,8-methano-4(*3H*)-quinazoline (**1**) was dissolved in acetonitrile (MeCN) and first the effect of the temperature was investigated. The results show that with 10 min residence time the best conversion value (86%) was obtained at 230 °C (Table 1, entry 4). It should be noted that at higher temperature, a significant amount of degradation product was observed and a brown oil was isolated (Table 1, entries 5 and 6). To further improve the conversion, the residence time was increased by utilizing longer coils (Table 1, entries 7 and 8). It was found that complete conversion can be obtained at 15 min residence time and the desired rDA product was isolated with 92% yield (Table 1, entry 7, Table 2, entry 1). With longer residence times, again, degradation of the product was observed. Importantly, the complete reaction parameter optimization was carried out only in 105 min. The parameters of the optimized reaction conditions and related results are summarized in Table 2.

**Table 2:** Comparison between batch reactions^a^ and the CF process for the synthesis of pyrimidinones **9**–**14**.

starting material	product	batch reaction (lit.)	CF in the present work			
		
		method: yield^c^ [%]	solvent^b^	temp [°C]	residence time [min]	yield^c^ [%]

**1**	**9**	A: 85 [47] B: 56 [46]	MeCN	230	15	92
**2a**	**9**	B: 54 [46] C: 85 [54]		250	10	95
**2b**	**9**	A: 63 [49] B: 58 [49] C: 72 [49]	toluene	230	30	93
**3**	**10**	B: 70–80 [50]	MeOH	130	10	95
**4**	**11**			150	10	97
**5**	**12**			120	10	95
**6**	**13**			130	10	94
**7**	**14**	B: 80 [45] C: 89 [54]	MeCN	210	15	96
**8a**	**14**	B: 80 [45]		220	10	96
**8b**	**14**	A: B: C: 0 [49] (no rDA occurred)		250	30	30^d^
**8b**	**15b**	–	EtOH/H_2_O = 2:1	250	30	90

Batch reaction^a^: Method A: reflux, CB, 12 h; Method B: performed at their melting points; Method C: MW, solvent: DCB (**2a**), EtOH (**7**), solvent-free (**8b**). ^b^Solvents were selected on the basis of solubilities. ^c^Isolated yield. ^d^After column chromatography.

In the case of di*-endo*-isomer **2a**, higher temperature (250 °C) but a shorter residence time was satisfactory to isolate **9** in a yield of 95%. Furthermore, we proceeded to investigate the elimination of cyclohexadiene from compound **2b**. Because of solubility reasons, the solvent was changed to toluene, which is known to be compatible with high-temperature conditions [51–53]. Retrodiene product **9** was afforded with full conversion and in an excellent yield of 93%, which is higher than the maximum yield (85%) reached in our previous batch work [54]. Importantly, this result was achieved with a residence time of 30 min.

Subsequently, tetracyclic methanopyrrolo-, methanopyrido- and methanoazepino[2,1-*b*]quinazolinones **3–6** were examined. Because of their excellent solubility, reactions were carried out in methanol (MeOH). Importantly, much milder reaction conditions gave satisfactory results. With the utilization of 120–150 °C and only 10 min residence time, full conversions and high yields (94–97%) were obtained. Lower yields were previously found (70–80%) using a batch process, even upon melting compounds **3**–**6** for 20 min [50].

The effect of the thioxo group on the rDA reaction was investigated too with compounds **7**, **8a** and **8b**. In the case of **7**, a yield of 96% was reached at full conversion at 210 °C in 15 min residence time. In the reaction of **8a**, the di-*endo* isomer of **7**, a slightly higher temperature was necessary, while an appropriate residence time of only 10 min was satisfactory to have **14** with 96% isolated yield.

**Scheme 2:**
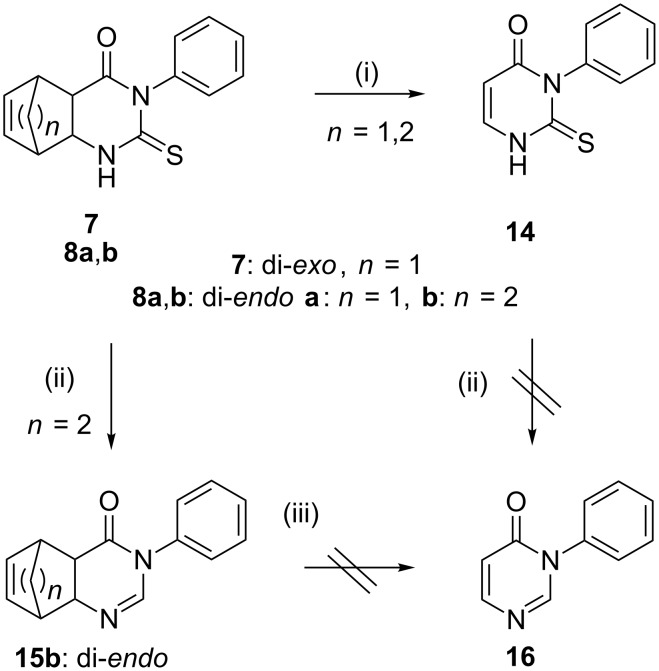
Synthesis of tricyclic ethanoquinazolin-4(*3H*)-one **15b**; (i) MeCN, *F*_R_ = 0.5 mL min^−1^, 220–250 °C; (ii) EtOH/H_2_O = 2:1, *F*_R_ = 0.5 mL min^−1^, 250 °C; (iii) MeCN, *F*_R_ = 0.5 mL min^−1^, 250 °C.

On the basis of these encouraging results, we decided to further examine the scope and limitation of the rDA reaction with the use of our CF reactor. Di*-endo*-3-phenyl-2-thioxohexahydro-5,8-ethanoquinazolin-4(*1H*)-one (**8b**) was selected, since this compound did not lose cyclohexadiene to form monocyclic **14** under batch and microwave conditions [49]. A solution of **8b** in MeCN was treated in the heated coil reactor at 250 °C, with a residence time of 30 min. Importantly, according to the HPLC–MS analysis, compound **8b** underwent thermal decomposition but only a moderate conversion (36%) was detected and **14** was isolated by means of column chromatography with a yield of 30%. This result is due to the lack of the quasi-aromatic character of the leaving cyclohexadiene, and possibly also due to the temperature limitation of our system. Surprisingly, however, we could detect traces of di*-endo*-3-phenyl-4a,5,8,8a-tetrahydro-5,8-ethanoquinazolin-4(*3H*)-one (**15b**), resulting from desulfurisation of **8b** (Scheme 2). This observation prompted us to investigate whether desulfurisation can occur under the flow reactor conditions. In the literature, a similar desulfurisation batch reaction was performed with nickel catalysis, in ethanol (EtOH)/water (2:1) solution [55–57]. Thus, thioxo derivative **8b** was dissolved in this mixture, and the CF method was repeated. Desulfurisation of **8b**, at 250 °C without adding any catalytic metal, provided tricyclic **15b** in good yield (90%). Most probably, the reaction was catalyzed by nickel, a component of the 304 stainless steel reactor coil [58–59]. These results also underline the importance to select appropriate solvents and tubing [60–61] for thermally driven reactions. In support of our results, tricyclic **15b** was also prepared alternately: the mixture of 3-aminobicyclo[2.2.2]oct-5-ene-carboxylic acid, triethylorthoformate, aniline and acetic acid was subjected to microwave irradiation at 120 W at 80 °C for 20 min. After completion of the reaction, as monitored by TLC, 20% methanolic solution in water was added to get precipitation. The solid was filtered off and washed with water to get di*-endo*-3-phenyl-4a,5,8,8a-tetrahydro-5,8-ethanoquinazolin-4(*3H*)-one (**15b**). All spectroscopic data of the alternately synthesized compound were the same as those obtained by the flow chemical method. The protocol for the synthesis of **15b** and the ^1^H and ^13^C NMR spectra of **15b** are shown in Supporting Information File 1 of this study.

A further attempt was made to perform the rDA reaction with **15b** at 250 °C with a residence time of 15 min in MeCN. However, the formation of **16** was not observed, that is the starting tetrahydroquinazolinone derivative **15b** did not undergo a thermally driven rDA reaction (Scheme 2). Furthermore, by applying the same conditions on **14**, no desulfurisation occurred and the formation of **16** was not detected either.

## Conclusion

In the case of compounds **1**–**8**, HPLC–MS measurements revealed full conversions to the desired pyrimidinones **9**–**14**, whereas only a moderate conversion of **8b** to **14** was observed. Mainly the retrodiene decomposition of compounds **1–8** occurred, since these latter possess the quasi-aromatic character, through the splitting-off of cyclopentadiene or cyclohexadiene. The stereochemistry (di-*endo* versus di*-exo* condensation) of the starting pyrimidinones **1**, **2**, **7** and **8** has no significant effect on the reaction yields. By using this safe, stable and scalable flow process, pyrimidinones **9**–**14** were afforded in high purity without the need for further purification steps. In addition, excellent yields and shorter reaction times are significant further advantages when compared to the corresponding batch processes. Moreover, the flow technology allowed the replacement of high-boiling and toxic solvents, which are commonly employed in batch process, e.g., CB or DCB, by less harmful, environmentally benign solvents such as toluene, MeCN, methanol, and ethanol.

In summary, we have developed a simple flow-based method for the preparation of pyrimidinone derivatives, precursors of a series of pharmacologically active materials, through the rDA reaction. The design of the reactor enabled accurate control of both residence time and reaction temperature. CF syntheses were performed under high-temperature conditions with varied solvents. The CF reactor set-up ensured enhanced safety and afforded yields higher than those for the batch and microwave processes. These could be achieved through careful reaction parameter optimization. It is particularly true for **8b**, which was unreactive under batch conditions, in contrast to a yield of 30% in CF. We envisage that this method can be readily extended to the preparation of other synthetically important building blocks requiring harsh conditions in batch methods. A simple, efficient and scalable production was implemented with a short processing time, which might open up new horizons for a potential CF industrial synthesis of heterocycles.

## Supporting Information

File 1 Experimental procedures and analytical data.
