# Supplementary material for: Continuous-flow retro-Diels–Alder reaction: an efficient method for the preparation of pyrimidinone derivatives
Source: Beilstein J Org Chem. 2018 Feb 1;14:318–24. doi: 10.3762/bjoc.14.20 (PMC5815275; doi:10.3762/bjoc.14.20)
Supplement: File 1 — Experimental procedures and analytical data. [file Beilstein_J_Org_Chem-14-318-s001.pdf]

## Supporting Information

## for

# Continuous flow retro-Diels–Alder reaction: an efficient method for the preparation of pyrimidinone derivatives

Imane Nekkaa[1], Márta Palkó[1], István M. Mándity[1], and Ferenc Fülöp*[1,2]

Address: [1]Institute of Pharmaceutical Chemistry, University of Szeged, Eötvös u. 6, H-6720 Szeged, Hungary, and [2]MTA-SZTE Stereochemistry Research Group, Hungarian Academy of Sciences, Eötvös u. 6, H-6720 Szeged, Hungary

Email: Ferenc Fülöp* - fulop@pharm.u-szeged.hu

*Corresponding author

## Experimental procedures and analytical data

**Contents**

1. Experimental procedures ........ S2

1.1. General information ........ S2

1.2. Procedures for the synthesis of the starting materials **1–8** ........ S2

1.3. General reactor set-up for continuous-flow reactions ........ S2

1.4. Additional table ........ S3

1.5. Syntheses and analytical data of the rDA Products **9–14** ........ S4

1.6. Desulfurisation reaction of **8b** ........ S23

1.6.1. Alternative batch synthesis of **15b** ........ S26

2. References ........ S26

## 1. Experimental procedures

### 1.1. General information

$^{1}H$ NMR spectra were recorded at 400.13 MHz or 500.20 MHz and the $^{13}C$-NMR spectra were recorded at 100.62 MHz or 125.77 MHz in CDCl3 at ambient temperature, with a Bruker AM 400 or Bruker Ascend™ 500 spectrometer. Chemical shifts are given in (ppm) relative to TMS as the internal standard. In the $^{1}H$ NMR spectra, signal positions (δ) are given in parts per million (ppm) from tetramethylsilane (δ = 0) and were measured relative to residual solvent signal ($CDCl_3$ = 7.26 ppm). $^{13}C$ NMR spectra were recorded using the same spectrometers. Signal positions (δ) are given in parts per million (ppm) from tetramethylsilane (δ = 0) and were measured relative to the signal of $CDCl_3$ (δ = 77.16 ppm). The multiplicities of signals are given as s = singlet, d = doublet, t = triplet, q = quadruplet, m = multiplet, ArH = aromatic. Coupling constants (*J*) are reported to the nearest 0.1 Hz. HPLC–MS measurements were performed with a Phenomenex 5 µm C18(2) 100 Å column (250 × 4.60 mm). The solvent system consisted of AcOH (0.1%) in water (A), AcOH (0.1%) in MeCN (B); gradient: 5%–100% B over 35 min, at a flow rate of 1 mL $min^{-1}$. Chromatograms and spectra were recorded in positive ionization mode with electrospray ionization on a Thermo LCQ Fleet mass spectrometer. FTIR spectrum was obtained from KBr pellet on a Perkin-Elmer 100 FTIR spectrometer.

### 1.2. Procedures for the synthesis of the starting materials 1–8

Starting materials **1-8** were synthesized in our previous work by the cyclization of the di-*exo*- or di-*endo*-norbornene-β-amino acids or esters with ethyl *p*-chlorobenzimidate resulting in tricyclic pyrimidinones **1**, **2a** and **2b** [1-4]. Methanopyrrolo-, methanopyrido- and methanoazepino[2,1-*b*]-quinazolinones **3-6** were prepared from di-*exo*-norbornene-fused azetidinone with lactim ethers by ring enlargement [5]. For the preparation of 2-thioxo-pyrimidinones **7**, **8a** and **8b**, the most common method is the reaction of the appropriate amino esters with phenyl isothiocyanate, followed by cyclization of the resulting thiourea with hydrogen chloride under reflux [1,4,6].

### 1.3. General reactor set-up for continuous-flow reactions

The modular flow system was equipped with a heated 304 stainless steel tubing coil with 14 mL internal volume [Supelco premium grade 304 empty stainless steel tubing; dimensions: length (L) x outer diameter (OD) x inner diameter (ID) = 100 ft x

1/16 in x 0.03 in; product reference number: 20553] and an adjustable back-pressure regulator (ThalesNano, BPR, 0–300 bar). The tube reactor was heated in a Heraeus oven to the desired temperature. Solutions of the starting materials **1–8** (0.1 mmol) were loaded into the reactor via an HPLC pump. The residence time was measured according to literature methods [7]. The resulting products **9–14** were identified by means of HPLC–MS, $^{1}H$ NMR and $^{13}C$ NMR spectroscopic analysis without the need for further purification steps. All physical and spectroscopic data of pyrimidinones **9–14** were identical with their literature data [1-6].

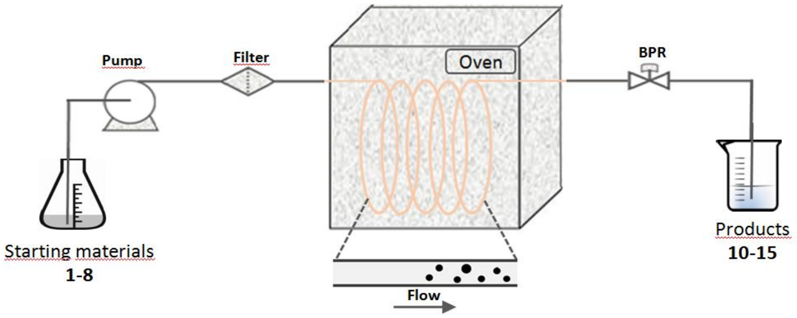


**Figure 1:** Schematic outline of the continuous flow reactor.

## 1.4. Additional Table

**Table S1:** Operating conditions for the rDA reactions in the CF reactor

| Entry | Product | Continuous flow in the present work | | | | |
|---|---|---|---|---|---|---|
| | | Solvent | Temp [ °C] | Residence time [min] | Flow rate [mL min$^{-1}$] | Yield[b] [%] |
| **1** | **9** | MeCN | 250 | 15 | 0.5 | 92 |
| **2a** | **9** | | 250 | 10 | | 95 |
| **2b** | **9** | Toluene | 230 | 30 | | 93 |
| **3** | **10** | MeOH | 130 | 10 | | 95 |
| **4** | **11** | | 150 | 10 | | 97 |
| **5** | **12** | | 120 | 10 | | 95 |
| **6** | **13** | | 130 | 10 | | 94 |
| **7** | **14** | MeCN | 210 | 15 | | 96 |
| **8a** | **14** | | 220 | 10 | | 96 |
| **8b** | **14** | | 250 | 30 | | 30[a] |
| **8b** | **15b** | EtOH/$H_2O$ = 2:1 | 250 | 30 | | 90 |

[a]After column chromatography
[b]Isolated yield

### 1.5. Syntheses and analytical data of rDA products 9–14

**2-(4-Chlorophenyl)-*3H*-pyrimidin-4-one** (**9**)

Synthesized from starting materials **1** (Method A), **2a** (Method B) and **2b** (Method C).

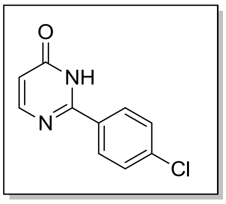


Method A

Di-*exo*-2-(4-chlorophenyl)tetrahydro-5,8-methano-4(*3H*)-quinazoline (**1**, 0.1 mmol) was dissolved in 10 mL of acetonitrile (MeCN). The system temperature was set to 250 °C, the pressure to 10 bar and the flow rate to 0.5 mL $min^{-1}$. When the pressure and the temperature of the flow system were stable, the solution loaded into the reactor passed through the heated reactor coil within a residence time of 15 min and the flow output was collected. The solvent was removed by evaporation and the resulting product **9** was analysed by means of HPLC-MS, $^{1}H$ NMR and $^{13}C$ NMR spectroscopy and obtained in a good yield and with high purity. (**9**, m.p. 243-245 °C, yield = 92%).

Method B

Di-*endo*-2-(4-chlorophenyl)tetrahydro-5,8-methano-4(*3H*)-quinazoline (**2a**, 0.1mmol) was dissolved in MeCN (6 mL) and reacted at 250 °C with a flow rate of 0.5 mL $min^{-1}$ corresponding to a residence time of 10 min. Product **9** was obtained by applying the same procedure used above, in a good yield and with high purity (**9**, m.p. 243-245 °C, yield = 95%).

Method C

Di-*endo*-2-(4-chlorophenyl)tetrahydro-5,8-ethano-4(*3H*)-quinazoline (**2b**, 0.01mmol) dissolved in toluene (15 mL) was introduced into the flow reactor at a decreased temperature of 230 °C while using the same flow rate (0.5 mL $min^{-1}$). The product mixture (brownish-yellow colour) leaving the flow system was collected and analysed by means of HPLC–MS spectroscopy. Based on the result obtained, **2b** showed a moderate conversion to **9**. Therefore, in order to get the complete conversion, we recycled the flow output twice. Product **9** was obtained within an overall residence

time of 30 min (**9**, m.p. 243-245 °C, yield = 93%).Spectroscopic data of **9** are in agreement with literature data [2-5].

$^{1}H$ NMR (400.1 MHz, $CDCl_3$) $\delta_H$: 6.45 (d, 1H, CH, *J* = 6.5 Hz), 7.53 (d, 2H, ArH, *J* = 8.5 Hz), 8.12–8.14 (m, 3H), 12.54 (s, 1H, NH).

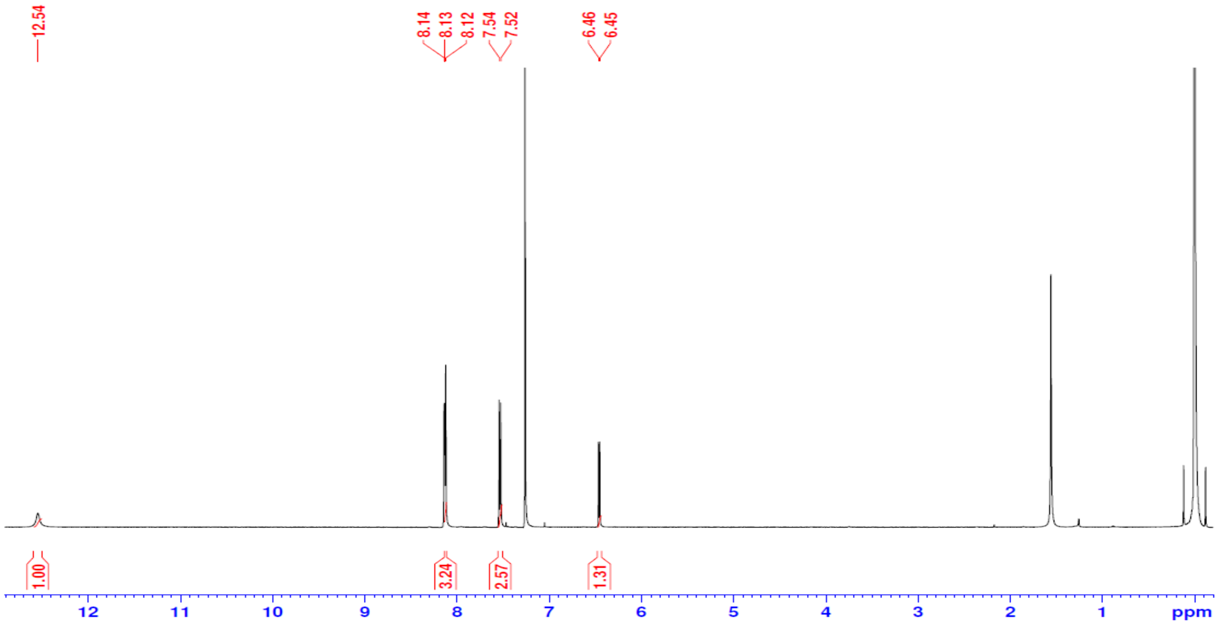


$^{13}C$ NMR (100.6 MHz, $CDCl_3$): $\delta_C$ = 113.84, 129.07, 129.45, 130.15, 138.81, 155.45, 156.65, 164.95.

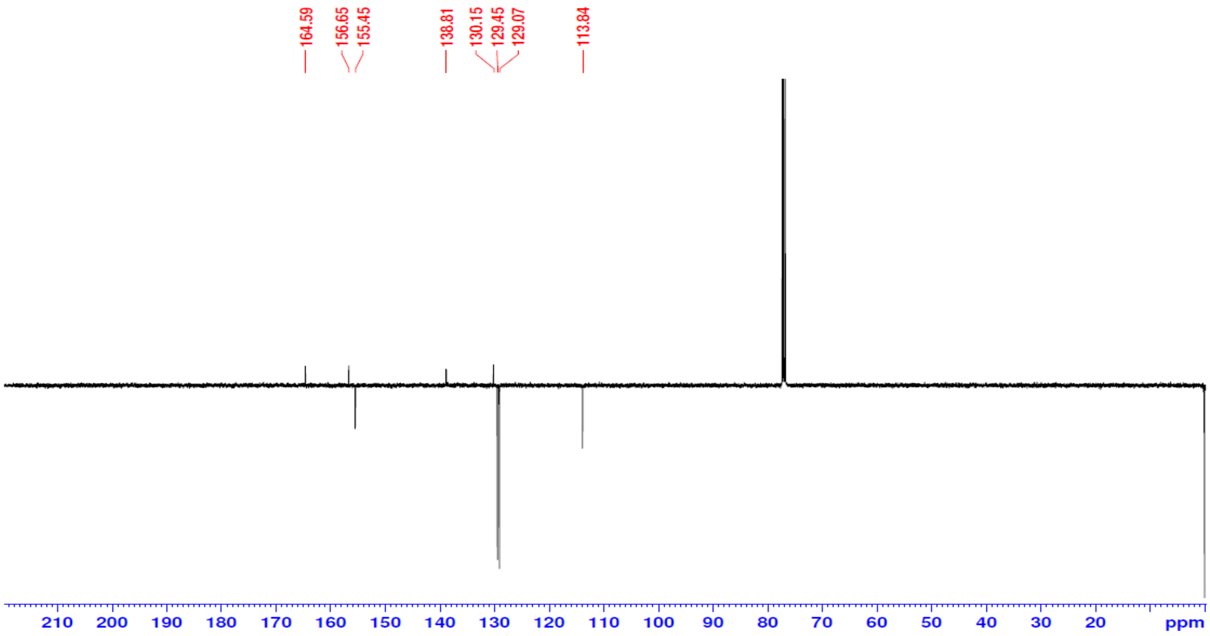

HPLC-MS characterization: The following retention time and molecular weight were determined: **9**: {RT= 15.16, m/z = 207 $[M + H]^+$ }.

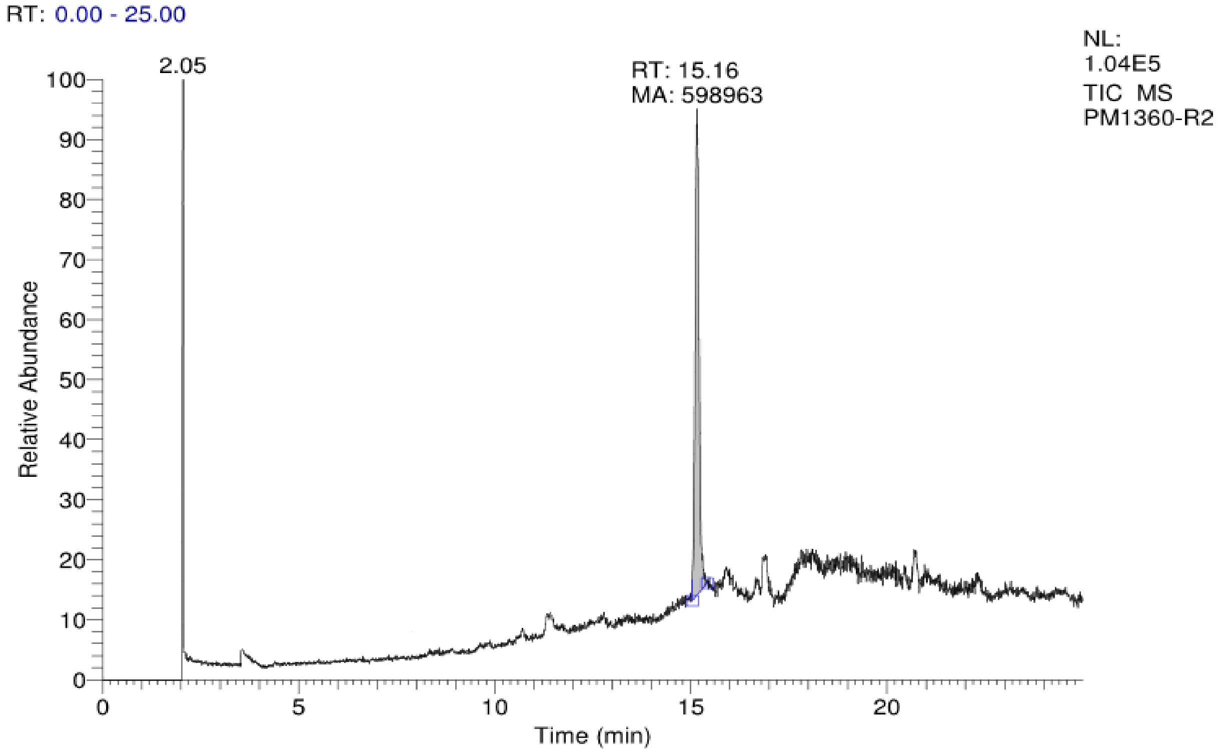


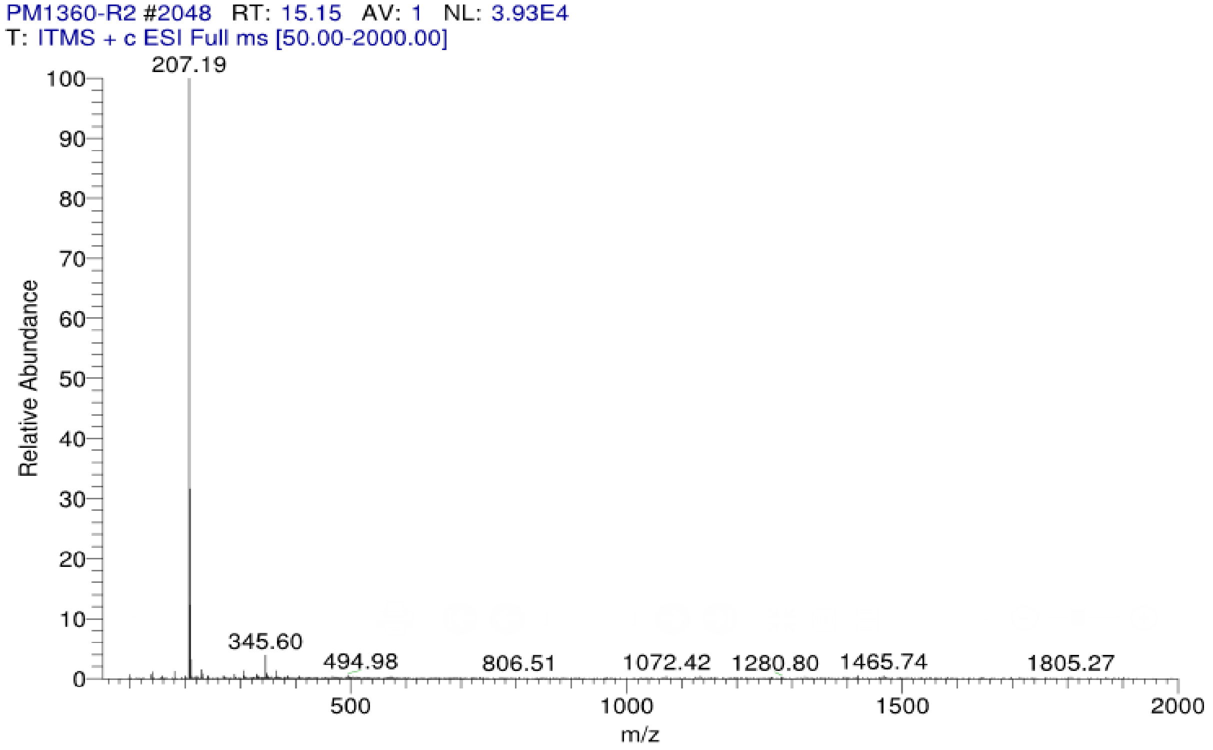

**7,8-Dihydropyrrolo[1,2-*a*]pyrimidin-4(*6H*)-one** (**10**):

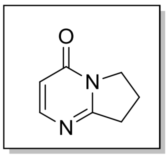


Di-*exo*-hexahydro-5,8-methanopyrrolo[2,1-*b*]quinazolin-9(*1H*)-one (**3**, 0.1 mmol) was dissolved in methanol (MeOH, 6 mL). The system temperature was set to 130 °C otherwise using the same pressure and flow rate as in previous procedures (10 bar, 0.5 mL min$^{-1}$). The solution fed into the reactor passed through the heated reactor coil within a residence time of 10 min and the flow output was collected. The solvent was removed by evaporation and the resulting product **10** was analysed and obtained in a nearly quantitative yield and with high purity (**10**, yield = 95%). Spectroscopic data are in agreement with the literature data [5].

$^1$H NMR (400.1 MHz, $CDCl_3$) $\delta_H$: 2.23–2.29 (m, 2H, $CH_2$), 3.12–3.15 (t, 2H, $CH_2$ *J* = 7.6 Hz), 4.15–4.17 (t, 2H, $CH_2$ *J* = 7.6 Hz), 6.28 (d, 1H, CH, *J* = 7.6 Hz), 7.86 (d, 1H, CH, *J* = 6.6 Hz).

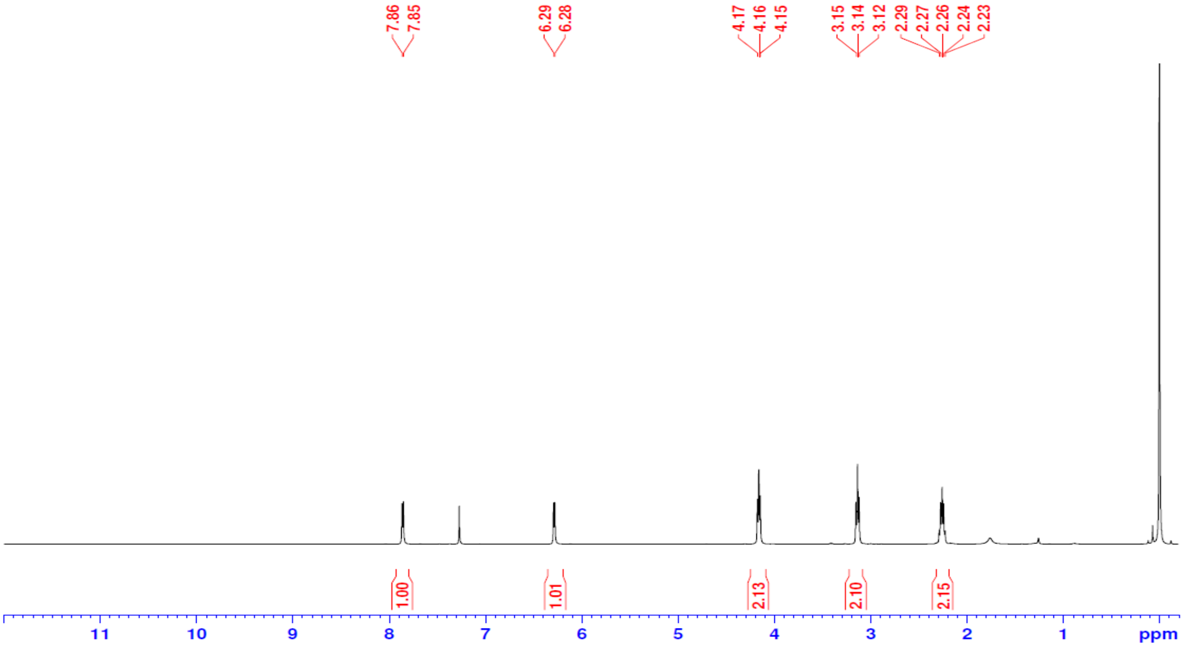

$^{13}C$ NMR (100.6 MHz, $CDCl_3$): $\delta_C$ = 18.90, 32.74, 47.01, 112.71, 154.15, 161.35, 164.73.

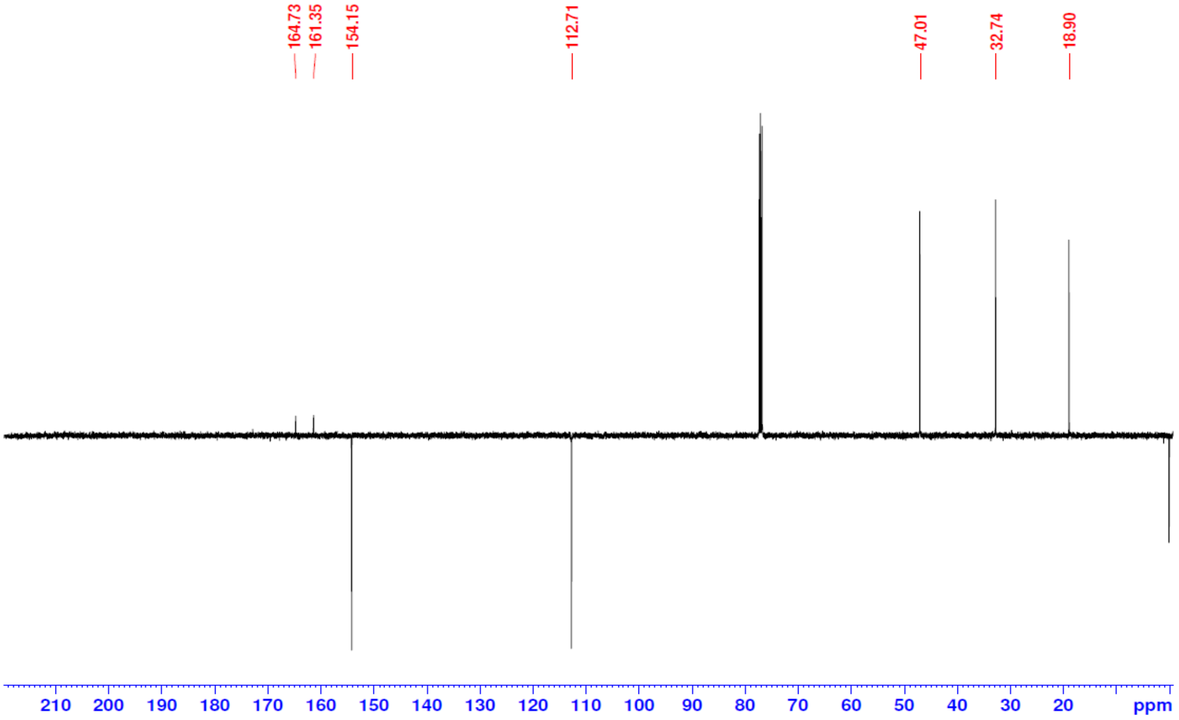

HPLC-MS characterization: The following retention time and molecular weight were determined: **10**: {RT= 2.60, m/z = 137 [M + H]$^+$ }.

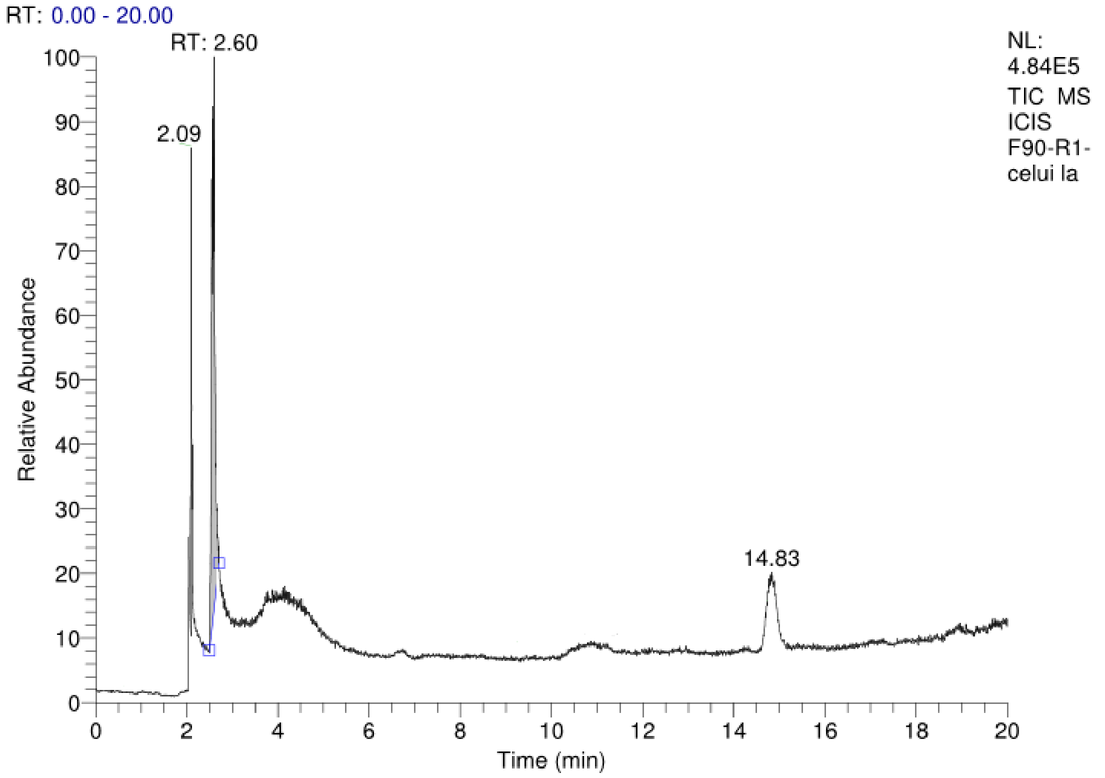


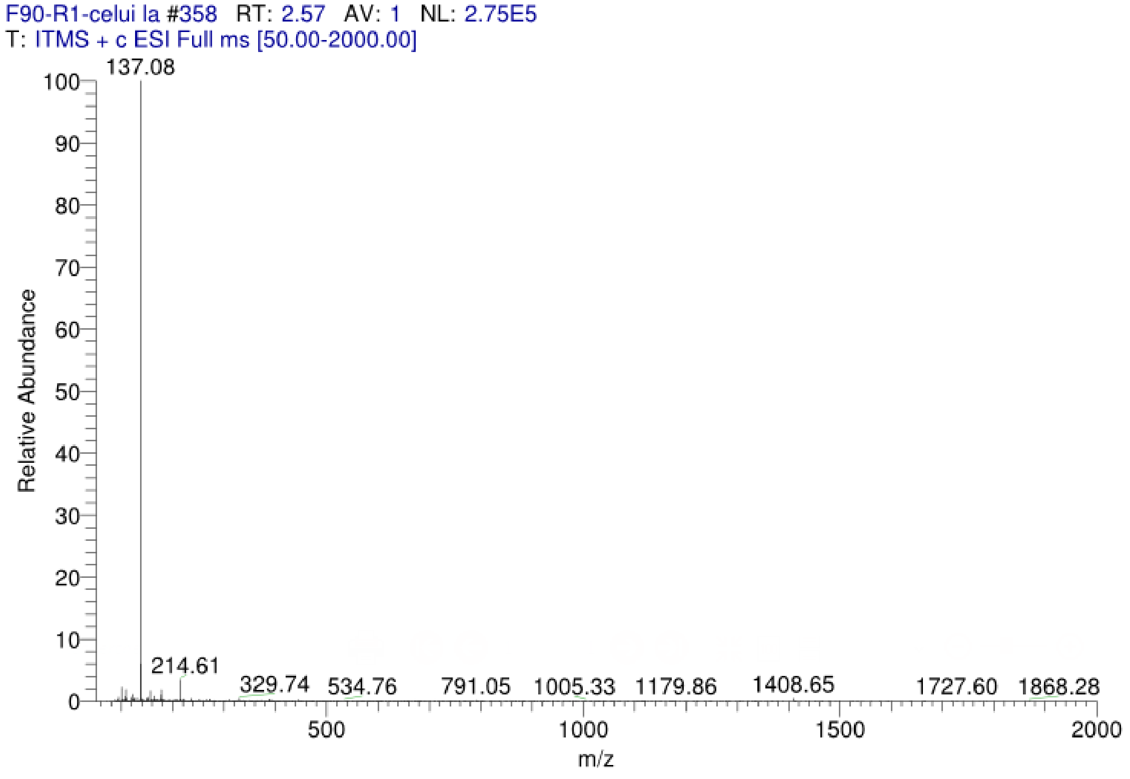

**6-Methyl-7,8-dihydropyrrolo[1,2-*a*]pyrimidin-4(*6H*)-one** (**11**)

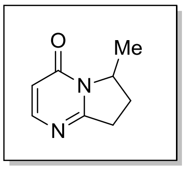

1-Methyl-hexahydro-5,8-methanopyrrolo[2,1-*b*]quinazolin-9(1*H*)-one (**4**, 0.1 mmol) was dissolved in MeOH (6 mL) at 150 °C, pressure = 10 bar, flow rate = 0.5 mL $min^{-1}$. The solution fed into the reactor passed through the heated reactor coil within a residence time of 10 min and the flow output was collected. The solvent was removed by evaporation and the resulting product **11** was analysed and obtained in a high yield and with high purity (**11**, yield = 97%).
Spectroscopic data are in agreement with the literature data [5].

$^{1}H$ NMR (400.1 MHz, $CDCl_3$) $\delta_H$: 1.47 (d, 3H, $CH_3$, *J* = 6.5 Hz), 1.86–2.44 (m, 2H, $CH_2$), 2.79–3.26 (m, 2H, $CH_2$), 4.77–4.84 (m, 1H, CH), 6.26 (d, 1H, CH, *J* = 6.6 Hz), 7.83 (d, 1H, CH, *J* = 6.4 Hz).

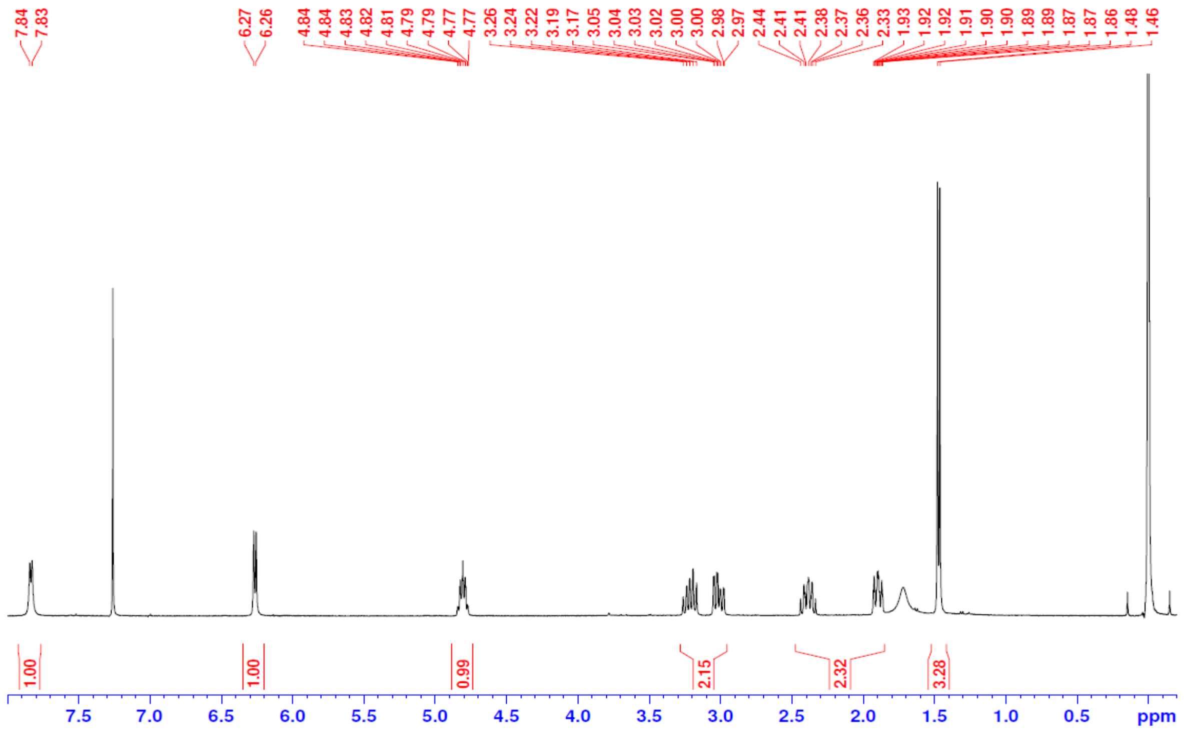

$^{13}C$ NMR (100.6 MHz, $CDCl_3$): $\delta_C$ = 18.72, 26.78, 31.01, 55.96, 113.30, 154.03, 161.08, 164.20.

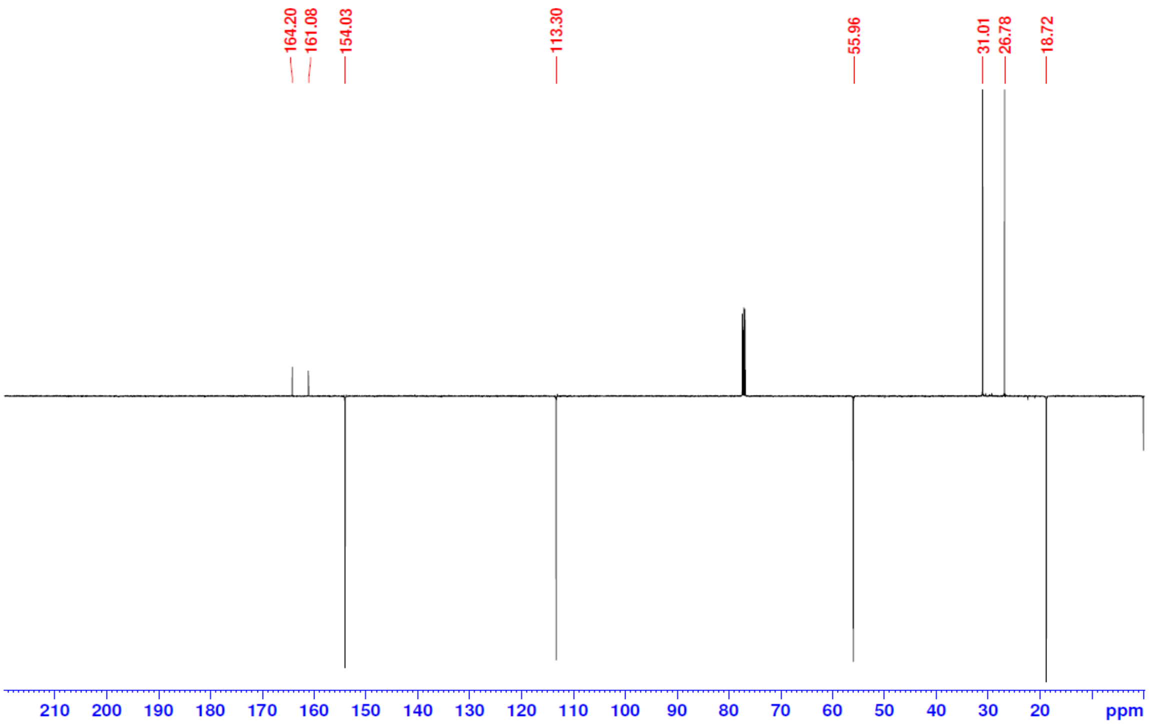

HPLC-MS characterization: The following retention time and molecular weight were determined: **11**: {RT= 2.96, m/z = 151 $[M + H]^+$ }.

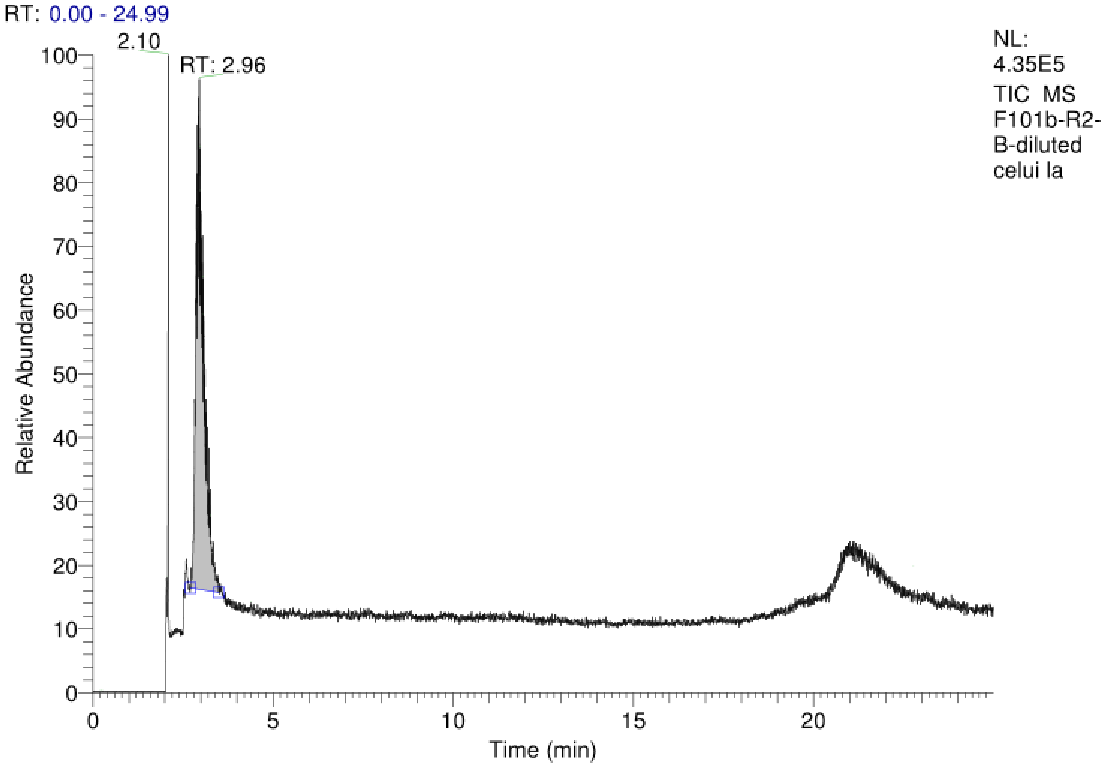


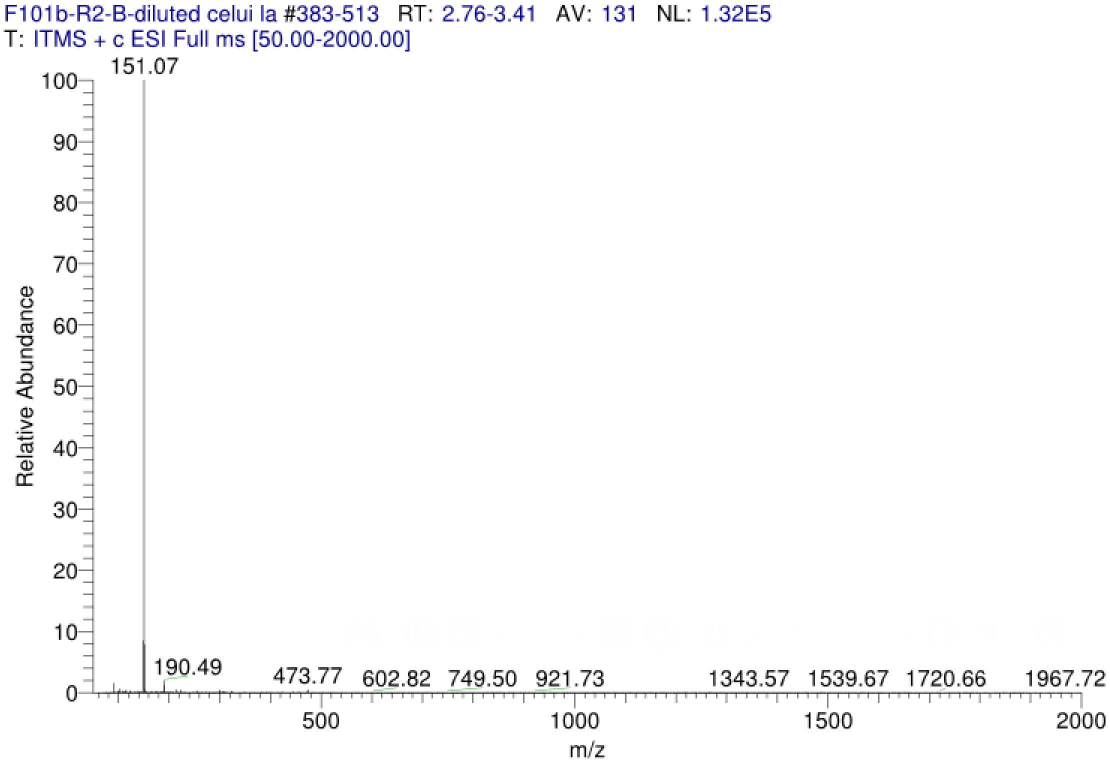

**6,7,8,9-Tetrahydropyrido[1,2-*a*]pyrimidin-4(*4H*)-one** (**12**):

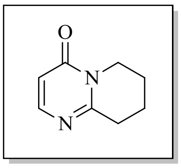

1,4-Hexahydromethanopyrido[2,1-*b*]quinazolin-11(11a*H*)-one (**5**, 0.1 mmol) was dissolved in MeOH (6 mL) at 120 °C, pressure = 10 bar, flow rate = 0.5 mL min$^{-1}$. The solution fed into the reactor passed through the heated reactor coil within a residence time of 10 min and the flow output was collected. The MeOH was removed by evaporation and the resulting product **12** was analysed and obtained in a high yield and with high purity (**12**, yield = 95%). Spectroscopic data are in agreement with the literature data [5].

$^{1}$H NMR (400.1 MHz, $CDCl_3$) $\delta_H$: 1.88–2.01 (m, 4H, $CH_2$), 2.91–2.94 (t, 2H, $CH_2$, *J* = 6.6 Hz), 3.95–3.98 (t, 2H, $CH_2$ *J* = 6.6 Hz), 6.31 (d, 1H, CH, *J* = 6.5 Hz), 7.81 (d, 1H, CH, *J* = 6.5 Hz).

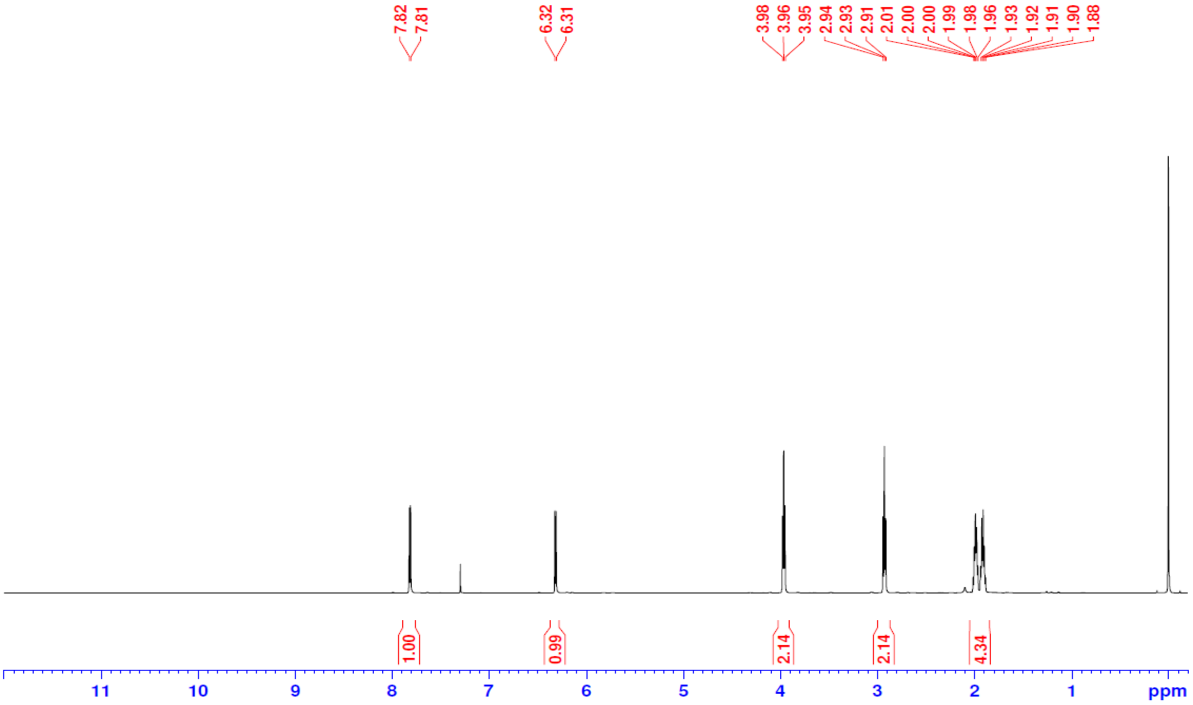

$^{13}C$ NMR (100.6 MHz, $CDCl_3$): $\delta_C$ = 19.12, 21.86, 31.68, 42.55, 112.25, 152.48, 160.33, 162.31.

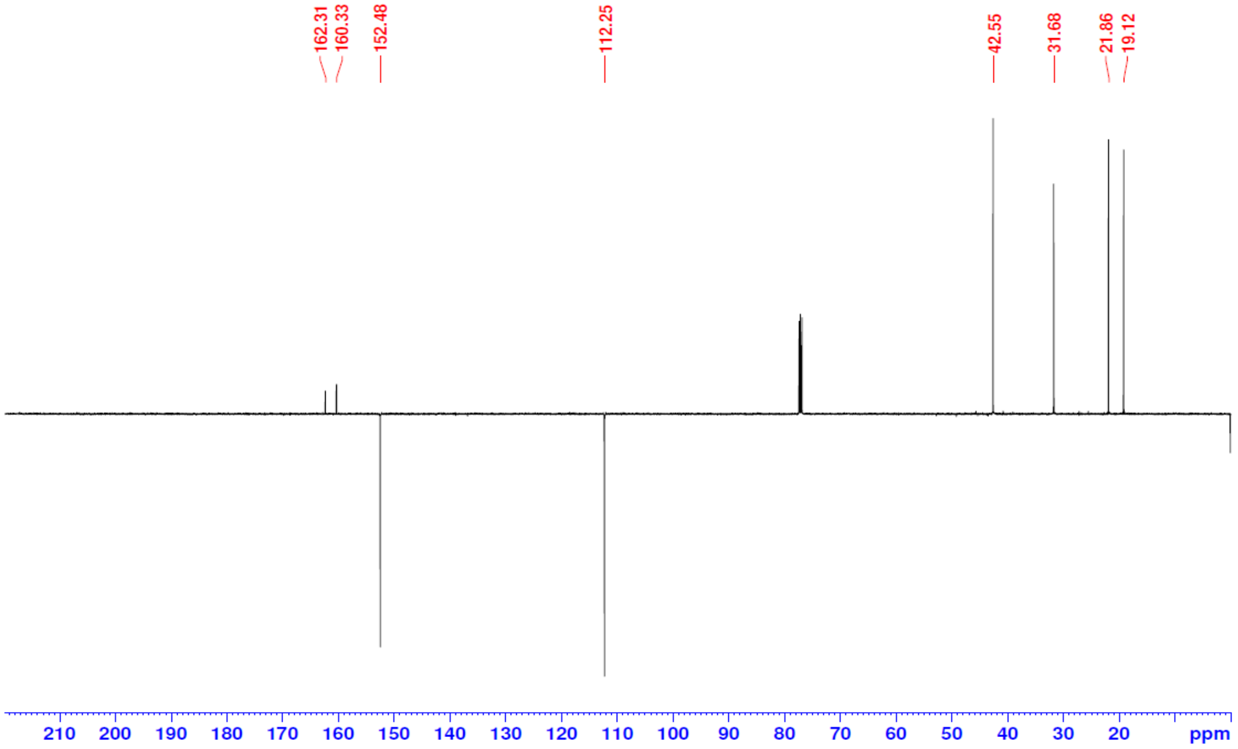

HPLC-MS characterization: The following retention time and molecular weight were determined: **12**: {RT= 7.67, m/z = 151 [M + H]$^+$}.

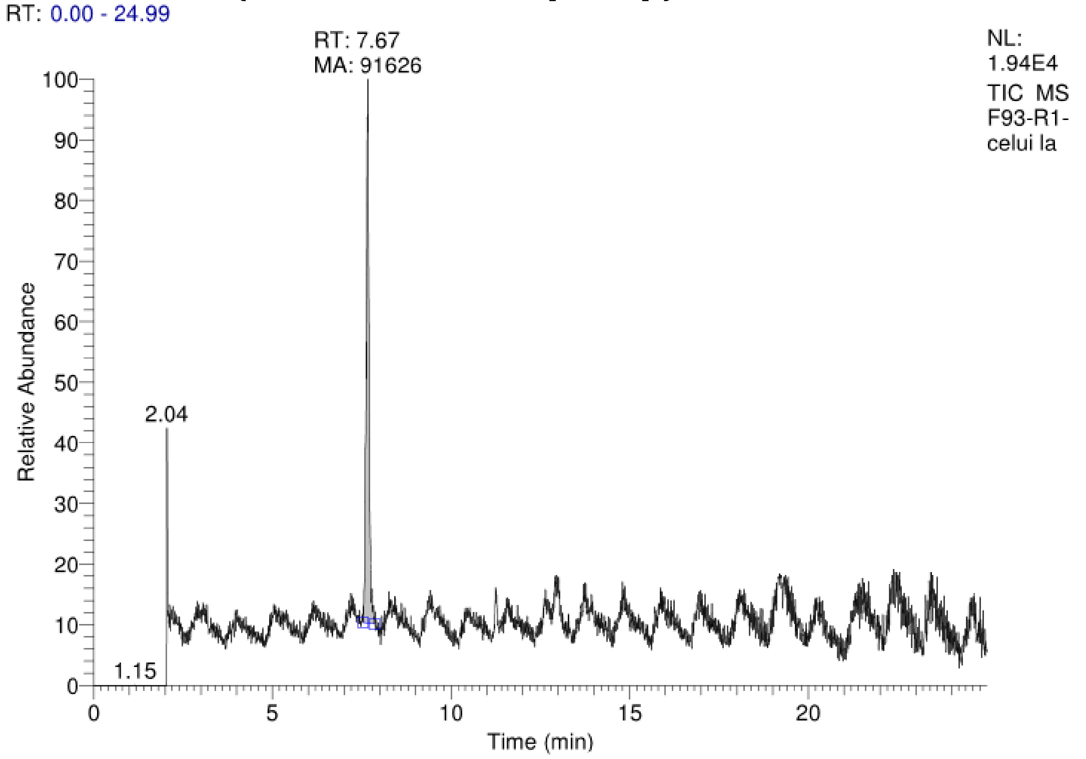


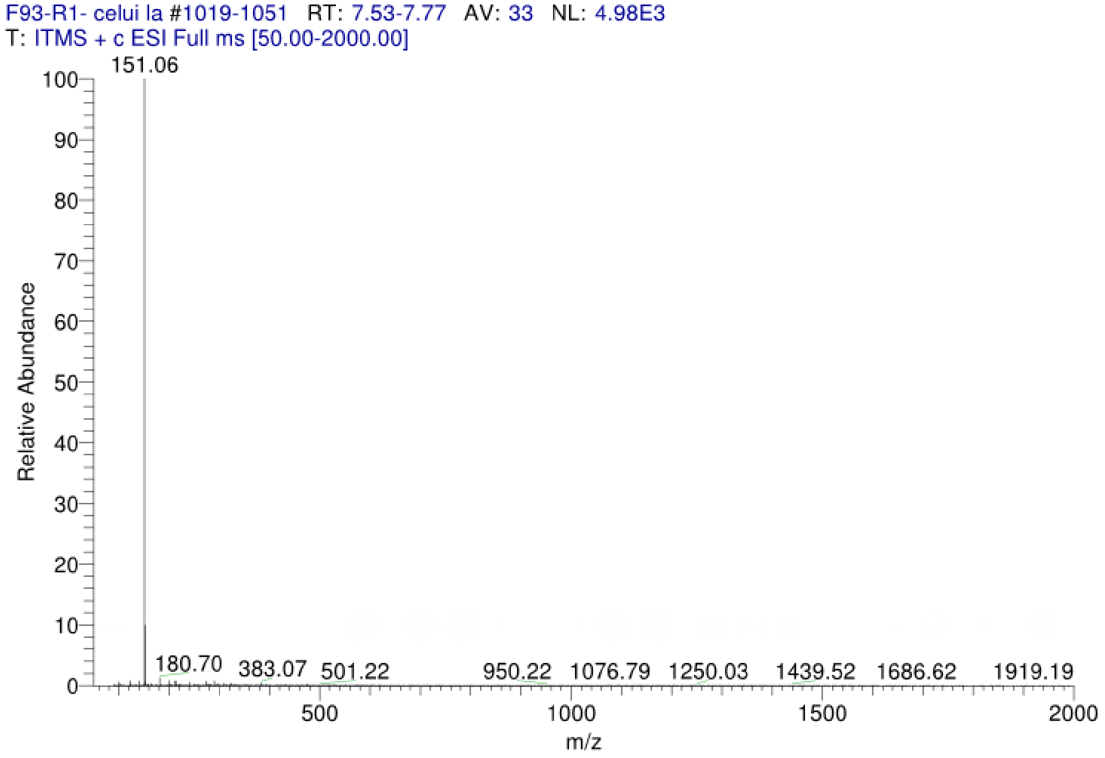

**7,8,9,10-Tetrahydropyrimido[1,2-*a*]azepin-4(*6H*)-one** (**13**):

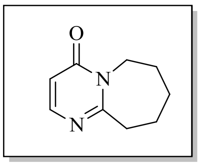

1,4-Octahydromethanoazepino[2,1-*b*]quinazolin-12(*6H*)-one (**6**) (0.1 mmol) was dissolved in MeOH (6 mL) at 130 °C, pressure = 10 bar, flow rate = 0.5 mL min$^{-1}$. The solution fed into the reactor passed through the heated reactor coil within a residence time of 10 min and the flow output was collected. The MeOH was removed by evaporation and the resulting product **13** was analysed and obtained in a good yield and with high purity (**13**, yield = 94%). Spectroscopic data are in agreement with the literature data [5].

$^1$H NMR (400.1 MHz, $CDCl_3$) $\delta_H$: 1.73–1.85 (m, 6H), 2.97–3.00 (m, 2H, $CH_2$), 4.30–4.33 (t, 2H, $CH_2$ $J$ = 6.5 Hz), 6.31 (d, 1H, CH, $J$ = 6.5 Hz), 7.74 (d, 1H, CH, $J$ = 6.5 Hz).

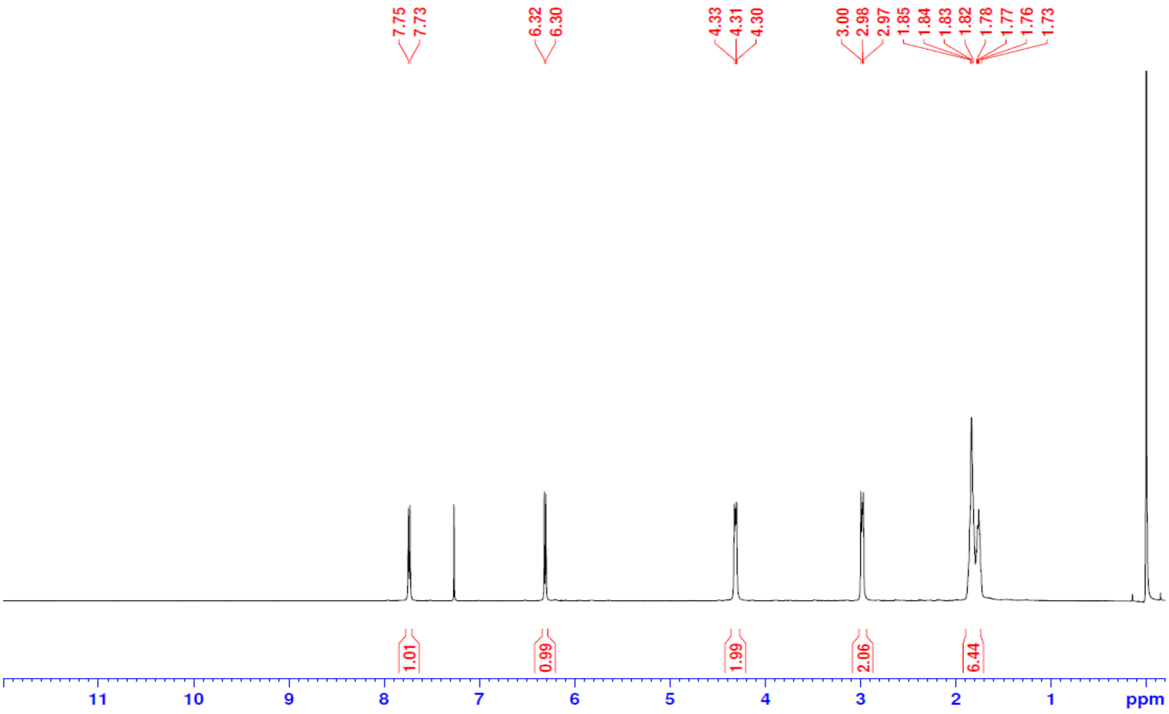

$^{13}C$ NMR (100.6 MHz, $CDCl_3$): $\delta_C$ = 24.71, 27.27, 29.70, 37.47, 42.70, 112.96, 152.34, 162.04, 165.24.

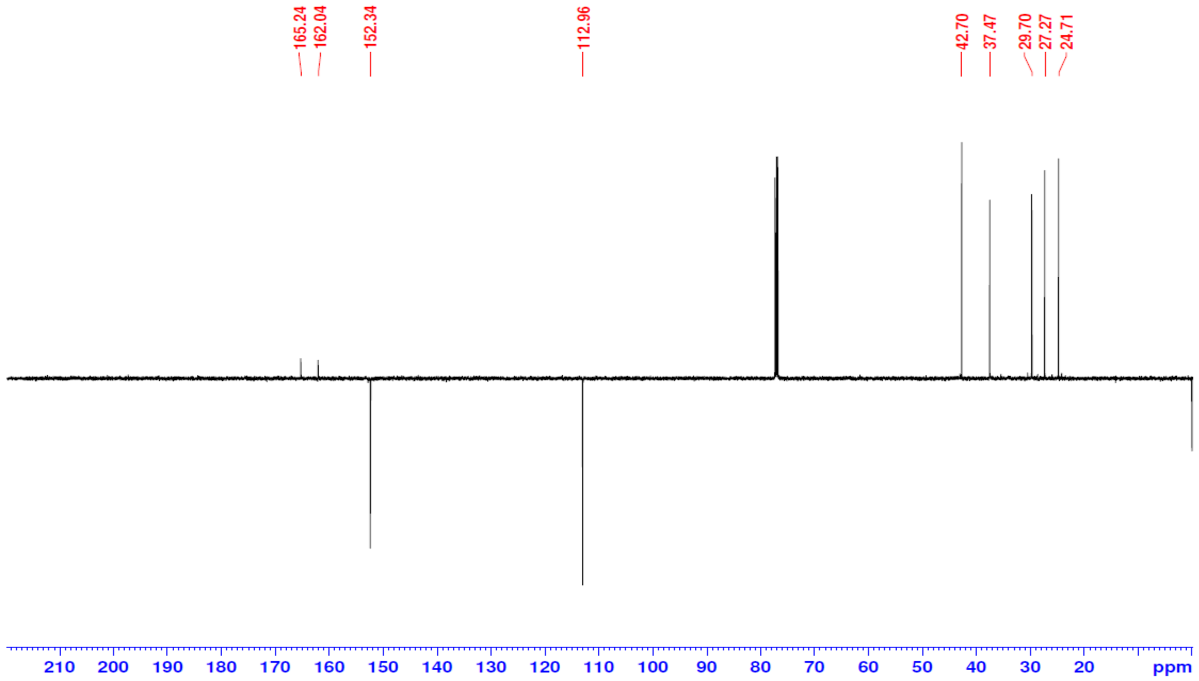

HPLC-MS characterization: The following retention time and molecular weight were determined: **13**: {RT= 11.66, m/z = 165 [M + H]$^+$}.

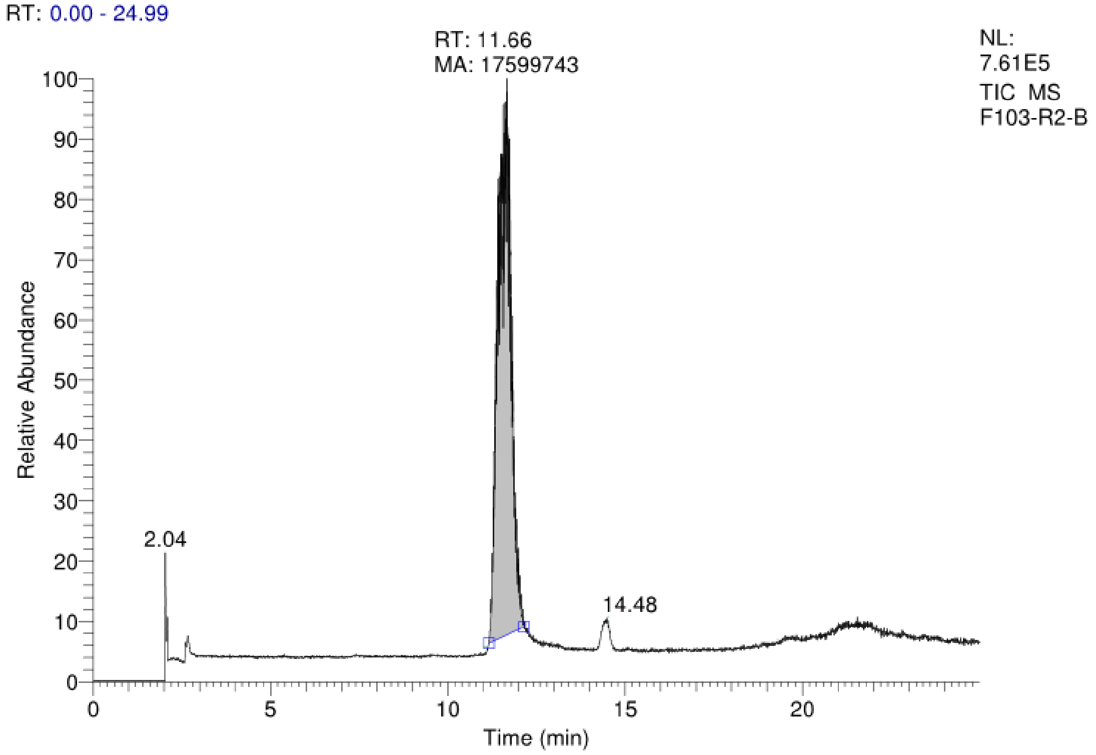


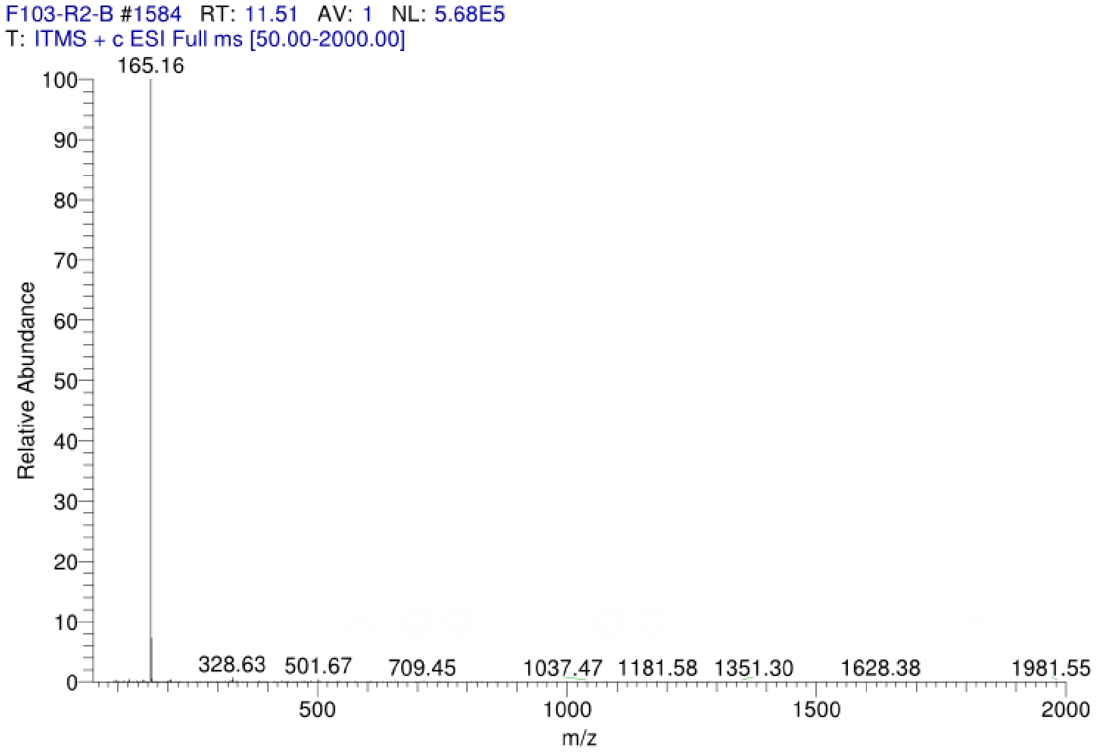

**2,3-Dihydro-3- phenyl-2-thioxopyrimidin-4(*1H*)-one (14)**

Synthesized from the following starting materials **7** (Method A), **8a** (Method B) and **8b** (Method C):

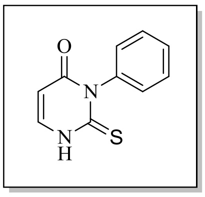


Method A

*diexo*-3-Phenyl-2-thioxohexahydro-5,8-methanoquinazolin-4(1*H*)-one (**7**, 0.1 mmol) was dissolved in MeCN (10 mL). The system temperature was set to 210 °C, the pressure to 10 bar and the flow rate to 0.5 mL $min^{-1}$. The solution loaded into the reactor passed through the heated reactor coil within a residence time of 15 min and the flow output was collected. The solvent was removed by evaporation and the resulting product **14** was analysed by means of HPLC-MS and $^1$H NMR and obtained in a high yield and with high purity (**14**, m.p. 247-249 °C, yield = 96%).

Method B

The synthesis was performed at 220 °C with a flow rate of 0.5 mL $min^{-1}$ corresponding to a residence time of 10 min. Di-*endo*-3-phenyl-2-thioxohexahydro-5,8-methanoquinazolin-4(1*H*)-one (**8a**, 0.1mmol) was dissolved in MeCN (10 mL). Product **14** was isolated by applying the same procedure used above (**14**, m.p. 247–249 °C, yield = 96%).

Method C

The system temperature was increased to 250 °C, while using the same flow rate (0.5 mL $min^{-1}$). Di-*endo*-3-Phenyl-2-thioxohexahydro-5,8-ethanoquinazolin-4(1*H*)-one (**8b**) (0.01 mmol) was introduced into the flow reactor in a solution of MeCN (15 mL). The solution fed into the reactor passed through the heated reactor coil within a residence time of 30 min and the flow output was collected and analysed. The solvent was removed by evaporation. The HPLC-MS chromatogram indicates that compound **8b** underwent thermal decomposition with only a moderate conversion of 36% to product **14**. The following retention times and molecular weights were determined: **8b**: {RT= 22.60, m/z = 285 $[M + H]^+$} **14**: {RT= 14.83, m/z = 205 $[M + H]^+$} Conversion: 36%.

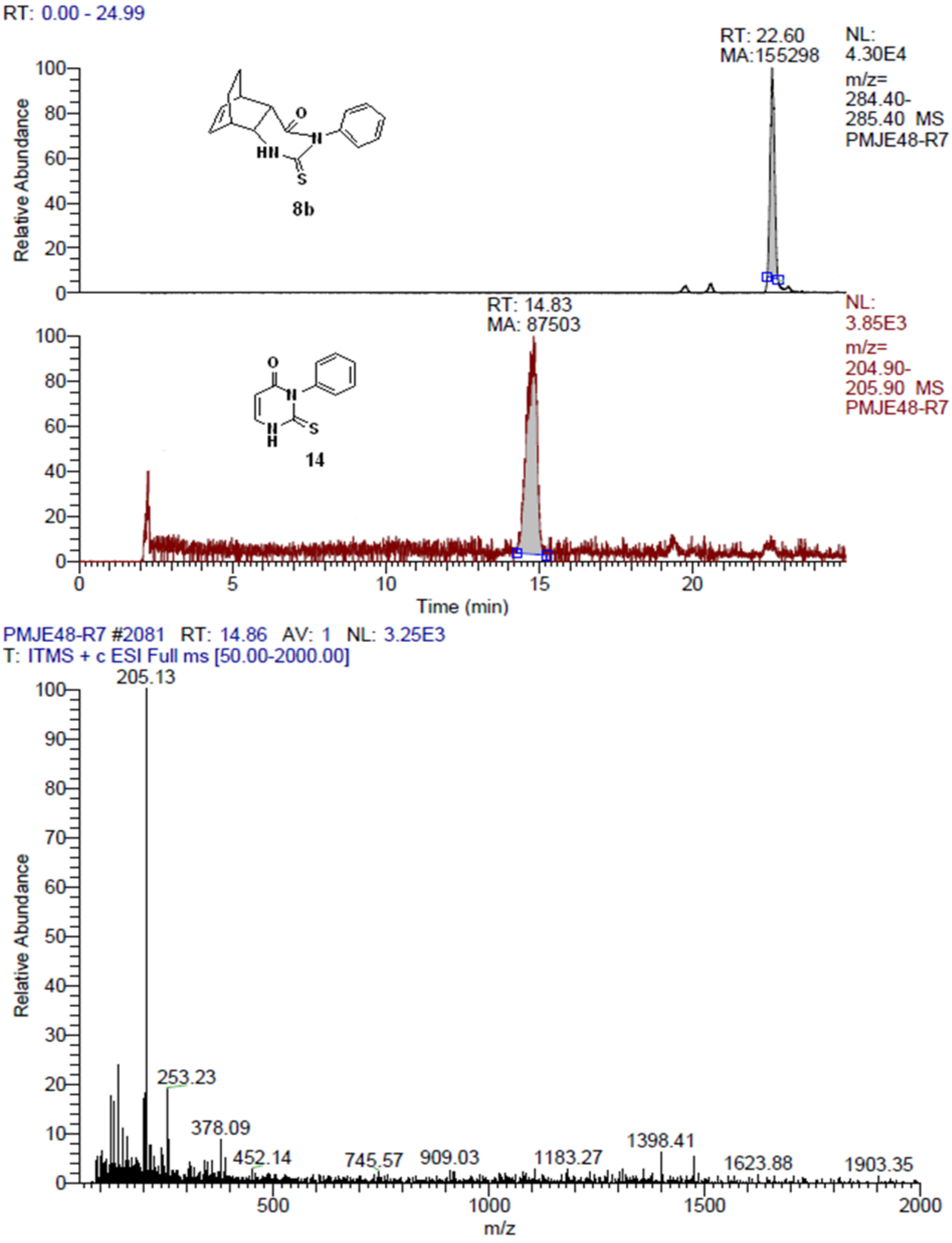


The processed product mixture leaving the flow system was subsequently loaded into a short silica chromatography column and eluted with a mixture of MeOH/toluene (1:1). The title compound was isolated as off-white crystalline material (**14**, m.p. 247-249 °C, yield = 30%). Spectroscopic data are in agreement with literature data [4,6].

$^1$H NMR (400.1 MHz, $CDCl_3$) $\delta_H$: 6.12 (d, 1H, CH, *J* = 7.6 Hz), 7.14-7.23 (m, 3H), 7.46–7.55 (m, 3H), 10.00 (s, 1H, NH).

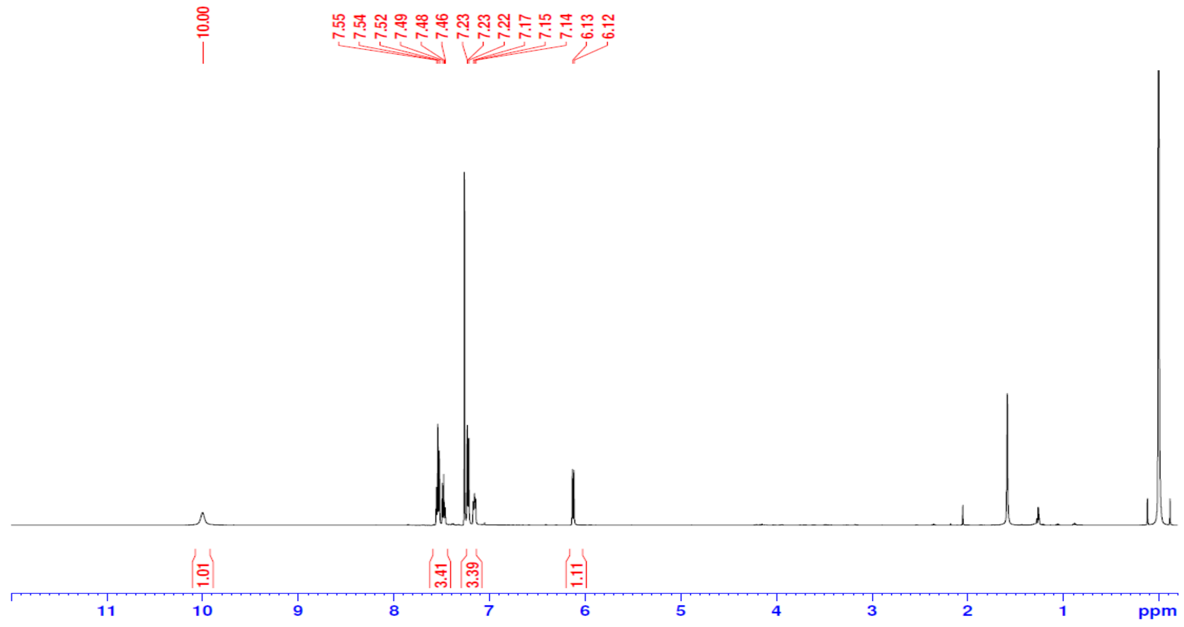


$^{13}$C NMR (100.6 MHz, $CDCl_3$): $\delta_C$ = 106.28, 127.92, 129.19, 129.75, 138.34, 138.78, 160.61, 178.43.

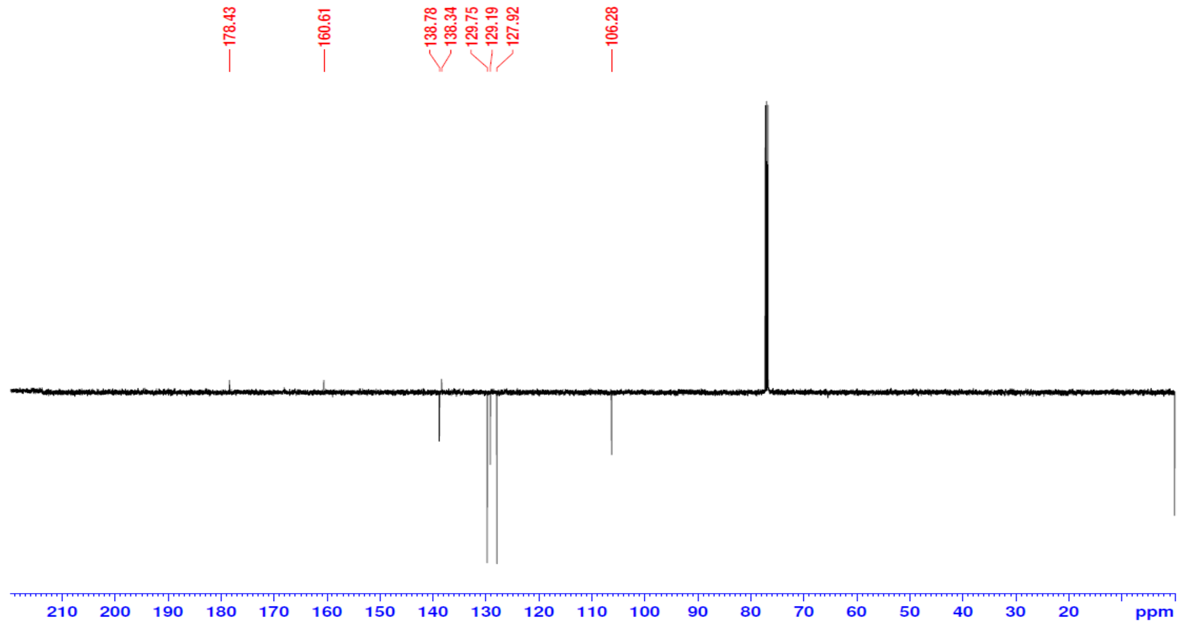

HPLC-MS characterization: The following retention time and molecular weight were determined: **14**: {RT= 13.78, m/z = 205 $[M + H]^+$}.

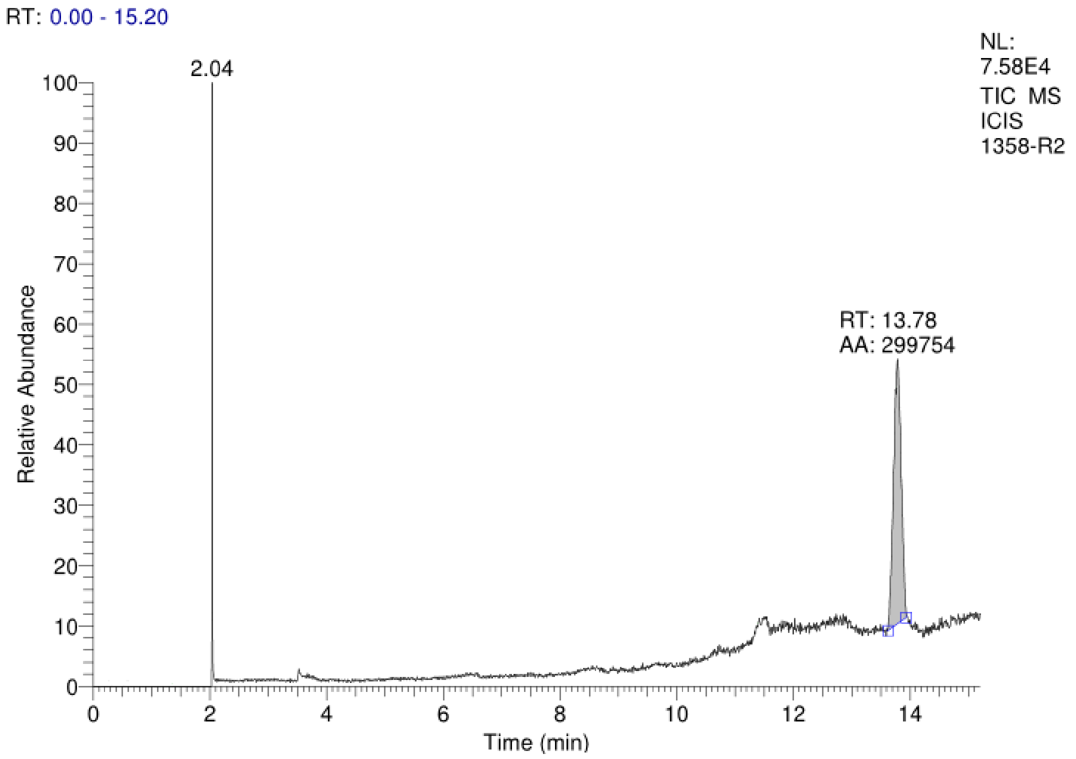


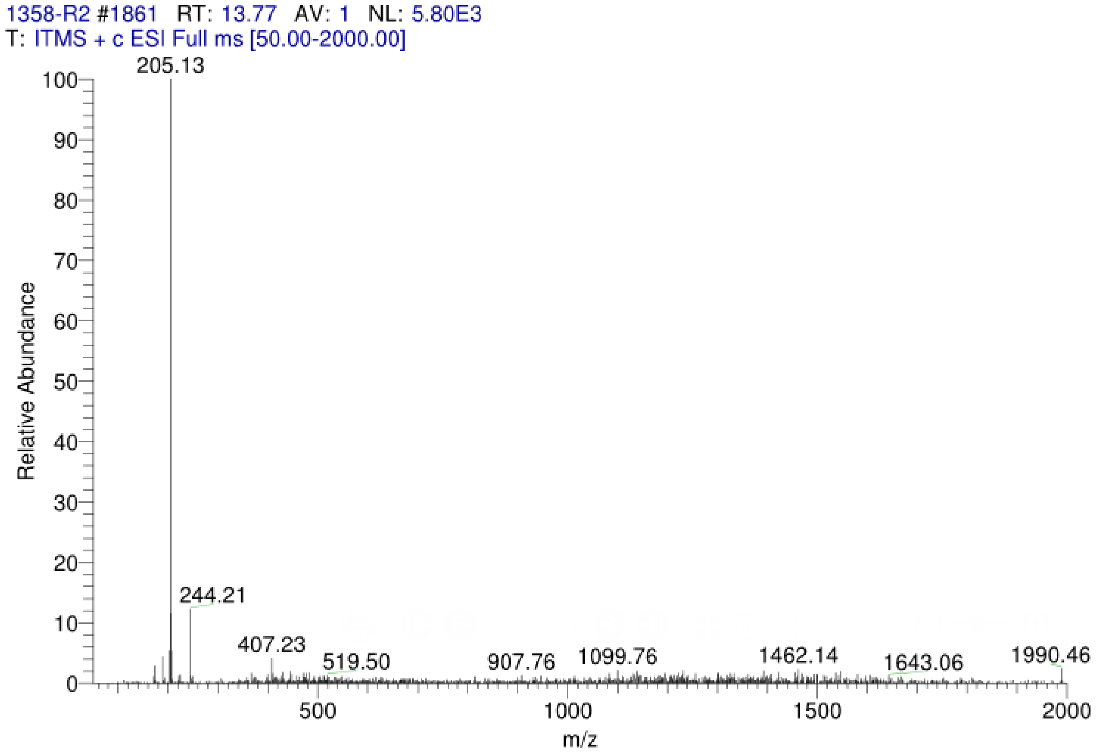

**1.6. Desulfurisation reaction of 8b, synthesis of *diendo*-3-phenyl-4a,5,8,8a-tetrahydro-5,8-ethanoquinazolin-4(3*H*)-one (15b)**

*diendo*-3-Phenyl-2-thioxohexahydro-5,8-ethanoquinazolin-4(*1H*)-one (**8b**, 0.01 mmol) was dissolved in a mixture of EtOH/water (2:1) (15 mL) at 250 °C, pressure = 10 bar, flow rate = 0.5 mL $min^{-1}$. The solution fed into the reactor passed through the heated reactor coil within a residence time of 30 min and the flow output was collected. Solvents were removed by evaporation, and the product residue was analysed. Product **15b** was obtained in a good yield (**15b**, m.p. 247-249, yield = 90%).

$^{1}$H NMR (400.1 MHz, $CDCl_3$) $\delta_H$: 1.21-1.51(m, 4H, $CH_2CH_2$), 2.83-2.97 (m, 3H, H-4a, H-5, H-8), 3.93 (d, 1H, H-8a, *J* = 8.5 Hz), 6.24-6.26 (t, *J* = 7.17, 1H, H-6), 6.79-7.00 (m, 6H, Ar, H-5), 7.81 (s, 1H, H-2).

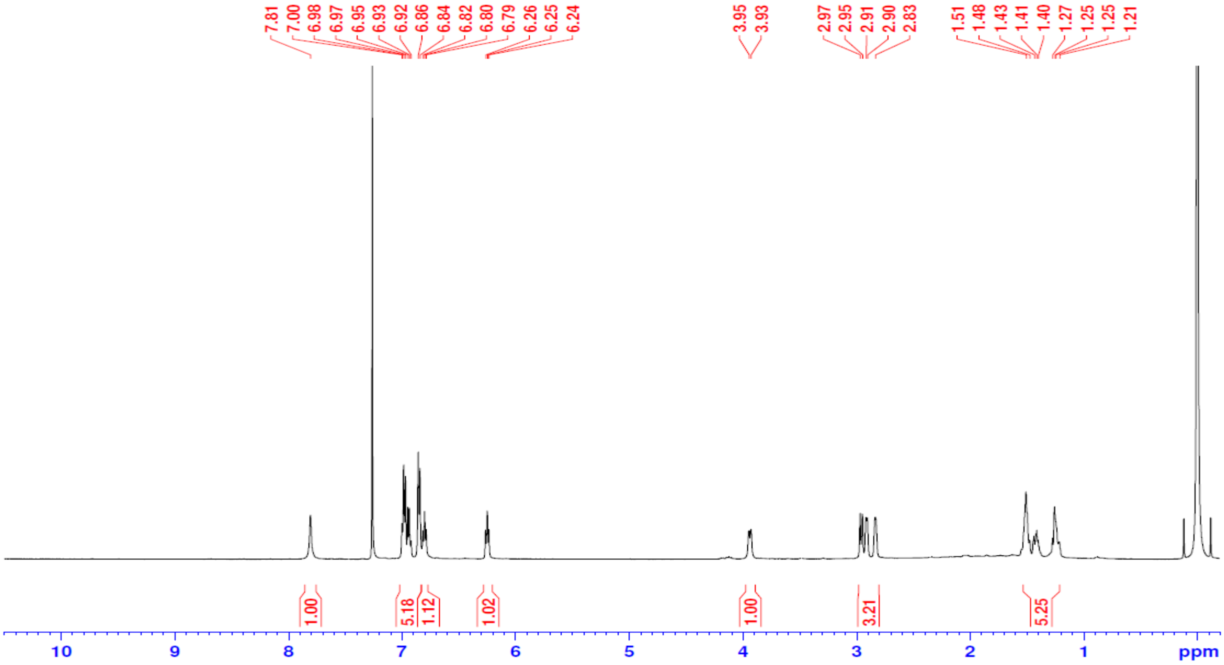

$^{13}C$ NMR (100.6 MHz, $CDCl_3$): $\delta_C$ = 22.54, 24.54, 32.38, 35.34, 54.43, 62.63, 118.18, 125.13, 125.86, 129.23, 138.21, 139.28, 153.27, 178.26.

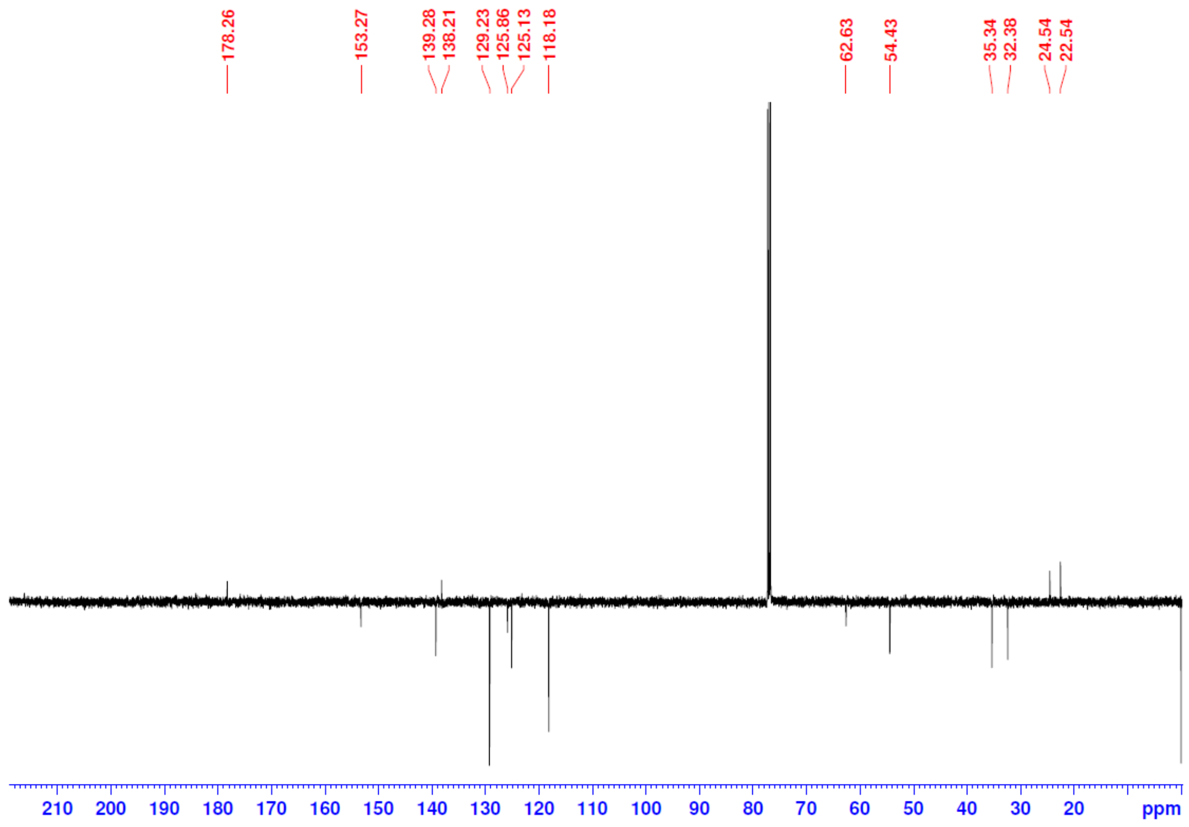

HPLC-MS characterization: The following retention time and molecular weight were determined: **15b**: {RT= 13.63, m/z = 253 [M + H]$^{+}$}.

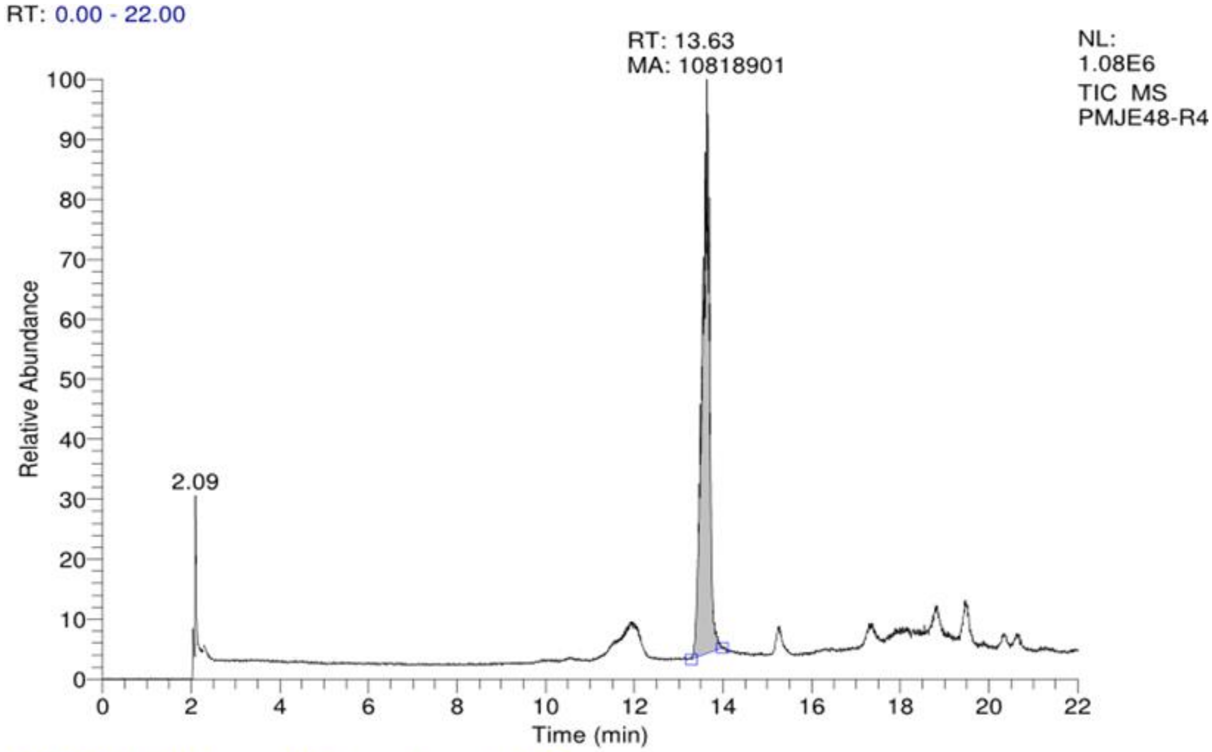


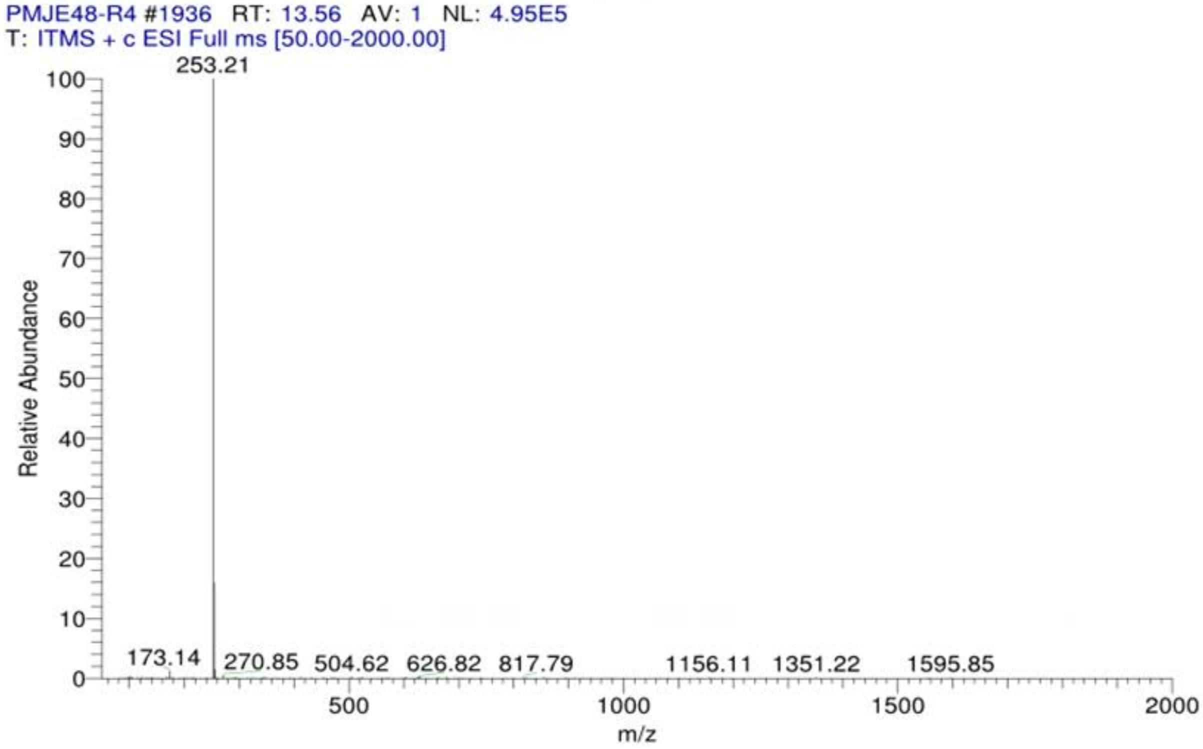

**1.6.1 Alternative batch synthesis of di-*endo*-3-phenyl-4a,5,8,8a-tetrahydro-5,8-ethanoquinazolin-4(3*H*)-one (15b)**

The mixture of 3-aminobicyclo[2.2.2]oct-5-ene-carboxylic acid (1 mmol, 167 mg), triethylorthoformate (1.5 mmol, 222 mg), aniline (1.1 mmol, 108 mg) and acetic acid (0.2 mL) was subjected to microwave irradiation at 120 W at 80 °C for 20 min. After completion of the reaction, as monitored by TLC, 20% methanolic solution in water (10 mL) was added to get precipitation. Solid was filtered off and washed with water to get **15b**. The product was purified by column chromatography on silica gel eluted with toluene/methanol (4:1). Yield: (163 mg, 65%); light pink crystals; m.p. 247–249 °C; IR: νC=O 1703, νC=N 1599 $cm^{-1}$ in KBr; $^{1}H$ NMR: 1.12-1.55(m, 4H, $CH_2CH_2$), 2.80-3.05 (m, 3H, H-4a, H-5, H-8), 3.93 (m, 1H, H-8a), 6.24 (t, $J$ = 7.17, 1H, H-6), 6.86-6.97 (m, 6H, Ar, H-5), 7.82 (s, 1H, H-2); $^{13}C$ NMR: 22.92, 24.95, 32.78, 35.76, 54.76, 62.90, 118.58, 125.59, 126.32, 129.66, 138.53, 139.65, 153.74, 178.68.

## References


1. Csende, F.; Stájer, G.; Fülöp, F. In: Comprehensive Organic Synthesis, 2nd ed. Elsevier, Amsterdam, 2014, vol. 5, pp. 551-592.
2. Stájer, G.; Szabó, A. E.; Fülöp, F.; Bernáth, G.; Sohár, P. *Chem. Ber.* **1987**, *120*, 259-264. doi: 10.1002/cber.19871200302
3. Stájer, G.; Szabó, A. E.; Bernáth, G.; Sohár, P. *Synthesis* **1987**, 290-292. doi: 10.1055/s-1987-27922
4. Palkó, M.; Sohár, P.; Fülöp, F. *Molecules* **2011**, *16*, 7691-7705. doi: 10.3390/molecules16097691
5. Fülöp, F.; Palkó, M.; Bernáth, G.; Sohár, P. *Synth. Commun.* **1997**, *27*, 195-203. doi: 10.1080/00397919708005019
6. Stájer, G.; Szabó, A. E.; Pintye, J.; Bernáth, G.; Sohár, P. *J. Chem. Soc.-Perkin Trans. 1.* **1985**, 2483-2487. doi: 10.1039/p19850002483
7. Ötvös, S. B.; Mándity, I. M.; Fülöp, F. *ChemSusChem*. **2012,** *5*, 266-296. doi: 10.1002/cssc.201100332
